# Chiral Nanocluster Complexes Formed by Host−Guest Interaction between Enantiomeric 2,6-Helic[6]arenes and Silver Cluster Ag_20_: Emission Enhancement and Chirality Transfer

**DOI:** 10.3390/molecules27123932

**Published:** 2022-06-19

**Authors:** Yan Guo, Ying Han, Chuanfeng Chen

**Affiliations:** 1 Beijing National Laboratory for Molecular Sciences, CAS Key Laboratory of Molecular Recognition and Function, Institute of Chemistry, Chinese Academy of Sciences, Beijing 100190, China; guoyan1101@iccas.ac.cn; 2 University of Chinese Academy of Sciences, Beijing 100049, China

**Keywords:** 2,6-helic[6]arene, silver cluster, host–guest interaction, luminescence, chirality transfer

## Abstract

A pair of chiral nanocluster complexes were formed by the host−guest interaction between the enantiomeric 2,6-helic[6]arenes and nanocluster Ag_20_. The formation and stability of the nanocluster complexes were experimentally and theoretically confirmed. Meanwhile, the chiral nanocluster complexes exhibited enhanced luminescence and induced CD signals at room temperature in the solid state, revealing the stable complexation and chirality transfer from the chiral macrocycles to the nanocluster Ag_20_.

## 1. Introduction

Metal nanoclusters (MNCs) have emerged as an excellent choice for constructing functional nanomaterials in recent years because of their disparate microscopic structures and unique macroscopic properties [1,2]. Hence, MNCs featuring strong chiral attributes and chiroptical activities [3,4] have been widely applied in asymmetric catalysis [5,6], negative refractive index materials [7], chemical sensing [8], optical materials [9], and biological imaging and therapy [10,11]. Therefore, the design and construction of novel chiral MNCs has gradually become a research focus in the field of nanomaterials [12,13].

Supramolecular assembly [14] has been extensively explored to modify the structures and morphology of MNCs, and even modulate their properties and functions. The application of macrocyclic hosts to construct metal nanocluster hybrid systems through host−guest assembly has gained great attention [15,16,17,18,19], especially macrocycle-based chiral MNC complexes. Zang and coworkers used the electrostatic assemblies between the crown ether−cation complexes and the chiral copper nanoclusters to modulate the chiral/achiral assembly and induce the circularly polarized luminescence of the nanocluster complexes for the first time [20]. Moreover, the specific chiral recognition between the chiral α-cyclodextrins and the nanoclusters could promote the enantio-separation of the racemic Au_20_ nanoclusters [21]. Pradeep and Purkayastha’s groups constructed chiral gold nanoclusters through host–guest assembly with chiral cyclodextrins and studied their chiroptical properties [22,23]. These results have revealed that constructing chiral MNCs assemblies with macrocyclic hosts can not only improve the stability of chiral MNCs, but also reversibly modulate the physicochemical properties and increase the diversity of chiral MNCs. However, it is still challenging to construct new macrocycle-directed chiral MNCs due to the deficiency of the synthetic chiral macrocycles.

**Figure 1:**
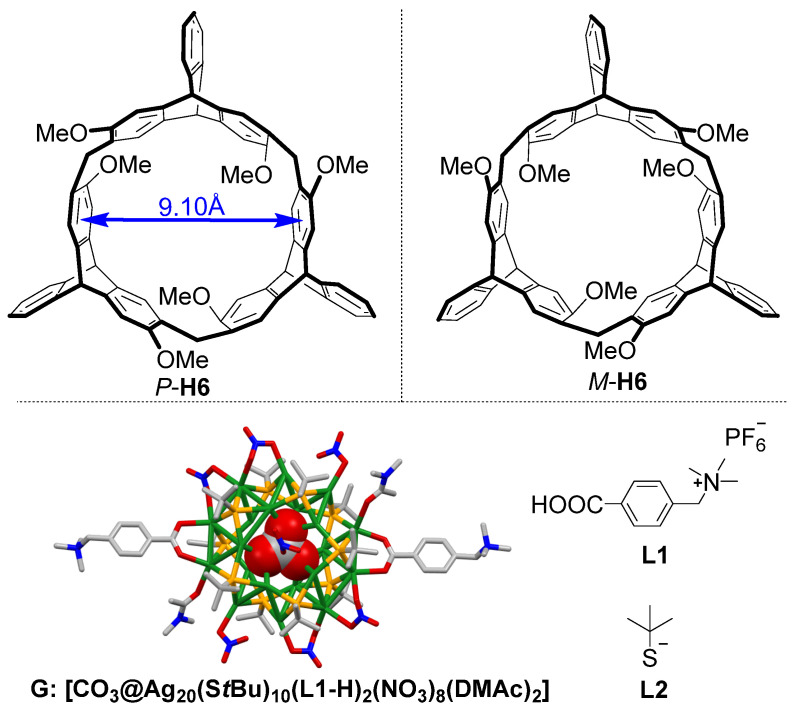
Structures of the chiral macrocyclic hosts *P*-**H6**/*M*-**H6**, the silver cluster **G**, and the ligands **L1** and **L2** (H atoms and solvent molecules of **G** were omitted for clarity; color legend: Ag, green; S, orange; C, gray; N, blue; O, red; **L1** was designated as **L1-H** after **L1** coordinated to Ag atoms with one proton lost).

In our previous work, we reported a new kind of chiral macrocyclic host, 2,6-helic[6]arene [24], which performed wide applications in molecular recognitions and self-assemblies [25,26,27,28,29]. Herein, we report a pair of new chiral nanocluster complexes (**G**@*P*/*M*-**H6**) formed by host−guest interactions between the chiral 2,6-helic[6]arenes (*P*/*M*-**H6**) and Ag_20_ nanocluster [30,31,32,33] **G** (Figure 1). It was found that the chiral nanocluster complexes showed enhanced emission and induced Cotton effects at room temperature, which indicated that encapsulation of the silver clusters by chiral 2,6-helic[6]arenes played an important role in modifying the properties of the silver clusters.

## 2. Results

### 2.1. Synthesis and Structures of the Silver Cluster

**Figure 2:**
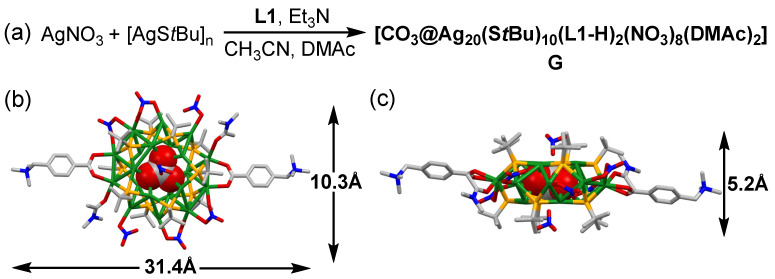
(**a**) The synthetic route of silver cluster **G**. (**b**) Top view and (**c**) side view of the crystal structure of **G**, H atoms and solvent molecules were omitted for clarity. Color legend: Ag, green; S, orange; C, gray; N, blue; O, red.

As shown in Figure 2a, **G** was synthesized by the mixing of AgNO_3_, precursor [AgS*t*Bu]_n_, ligand **L1**, and triethylamine (Et_3_N) in a mixed solvent of CH_3_CN and N,N-dimethylacetamide (DMAc) (*v*/*v* = 1:1) under vigorous stirring for 30 min at room temperature. The block crystals of **G** were obtained by slow vapor diffusion of tetrahydrofuran into the above solution in darkness. X-ray crystallographic analysis (Table S1) revealed that the solid-state structure of **G** (Figure 2b,c) was similar to the reported Ag_20_ nanoclusters [31,32,34], which possessed a drum-like Ag_20_S_10_ core with one CO_3_^2−^ anion in the center. Two trimethylammonium **L1-H** ligands were anchored to the Ag_20_S_10_ core in the antiparallel direction by the linking of the carboxylic groups and the peripheral silver atoms. Additionally, two DMAc and six NO_3_^−^ anions coordinated with the silver atoms around the silver clusters, and two remaining NO_3_^−^ anions covered the top and the bottom of the Ag_20_S_10_ core. The maximum outer diameter of **G** was ca. 31.4 Å, with a thickness of 5.2 Å.

### 2.2. Study on the Formation of the Nanocluster Complexes

It was reported that the cavity size of host *P*/*M*-**H6** is about 9.10 Å (Figure 1), which could form good binding with trimethylammonium groups through C–H∙∙∙π and cation–π interactions [35]. The host–guest interaction between *P*/*M*-**H6** and ligand **L1** was quantificationally investigated by Job plot and ^1^H NMR titration experiments (Figures S1–S4), confirming that *P*/*M*-**H6** and ligand **L1** could form stable complexes in a 1:1 binding mode. Based on the above results, the nanocluster complex **G**@*P*-**H6** was prepared by dissolving the host *P*-**H6** and the crystal of **G** in the molar ratio of 2:1 in the mixed solvent of CH_3_CN and DMSO (*v*/*v* = 5:1). After stirring for 30 min, ether was added to precipitate the **G**@*P*-**H6** complex. The turbid liquid was centrifuged, and its supernatant was discarded. Then, the white nanocluster complex was collected after the residual solvent was evaporated in 92.3% yield. The enantiomeric complex **G**@*M*-**H6** was synthesized by the same method in 89.9% yield.

**Figure 3:**
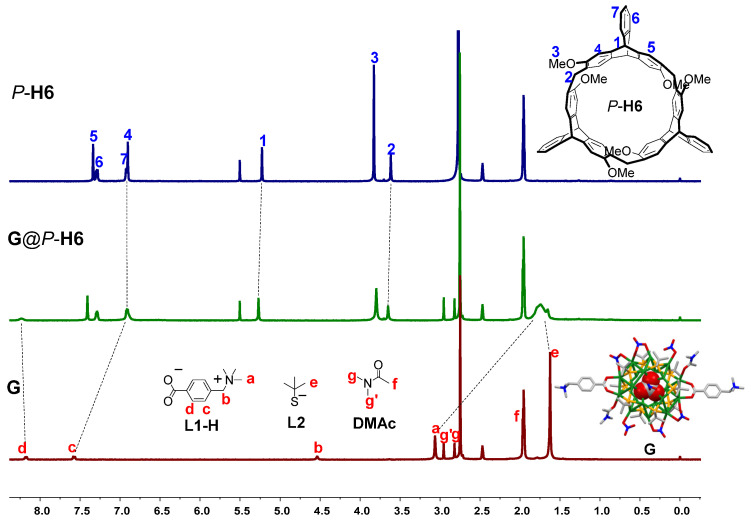
^1^H NMR spectra of *P*-**H6**, **G**, and **G**@*P*-**H6** in CD_3_CN:DMSO-*d_6_* = 5:1 (*v*/*v*) at 298 K (400 MHz, *P*-**H6** = 2.5 mM, **G** = 1.25 mM; blue and red symbols represent the proton signals of *P*-**H6** and **G**, respectively).

NMR spectroscopy was used to investigate the host−guest interaction of complex **G**@*P*-**H6** in solution. When the complex was dissolved 5:1 (*v*/*v*) in CD_3_CN/DMSO-*d_6_*, the ^1^H NMR spectrum of **G**@*P*-**H6** showed a distinctive difference from that of free *P*-**H6** and **G** (Figure 3), which demonstrated the formation of the complex. The trimethylammonium proton H_a_ on **L1-H** of **G** exhibited pronounced up-field shifts due to the shielding effect of the cavity of *P*-**H6**, and aromatic proton H_c_ also shifted up-field to 6.9 ppm, overlapping with the proton signals of H_4_ and H_7_. Meanwhile, the aromatic proton H_d_ on **L1-H** and proton H_e_ on the *t*-butyl mercaptan ligand **L2** of **G** shifted downfield because of the de-shielding effect of the methoxyl groups outside the host cavity. However, the signals related to the methylene proton H_b_ on **L1-H** disappeared after complexation, which could be caused by the encapsulation of the cavity of *P*-**H6** and the extensive broadening effects due to the complexation dynamics. The proton signals of H_1_ and H_2_ on *P*-**H6** only showed slight downfield shifts because of the complexation. These observations indicated that in complex **G**@*P*-**H6**, the trimethylammonium ligand **L1-H** of **G** was encapsulated into the cavity of the macrocyclic host, and the ligand **L2** of **G** was outside of the cavity. To further confirm the complexation between the host and the silver cluster, the 2D nuclear Overhauser enhancement spectroscopy (NOESY) NMR experiment was carried out (Figure S7). It was found that the NOE correlation signals between the proton H_1_ of *P*-**H6** and trimethylammonium proton H_a_ of **G** demonstrated the formation of the nanocluster complex in solution, which was consistent with the results of the ^1^H NMR spectra. The ^1^H and 2D NOESY NMR spectra of the nanocluster complex between *M*-**H6** and **G** are shown in Figures S6 and S8, suggesting the same complex capacity as that of *P*-configuration.

The solid-state ^13^C NMR experiments were also carried out to gain insight into the complexation of the nanocluster complexes in solid state. The signals of the solid-state ^13^C NMR spectrum showed that the peaks of carbons a–b on **L1-H** of **G** distinctly shifted up-field and C_1_–C_3_ of *P*-**H6** exhibited downfield changes (Figure S9), suggesting the good binding between the host and the silver cluster in solid state. Meanwhile, the similar signal changes of complex **G**@*M*-**H6** in Figure S10 demonstrated the same complexation as that of **G**@*P*-**H6**.

**Figure 4:**
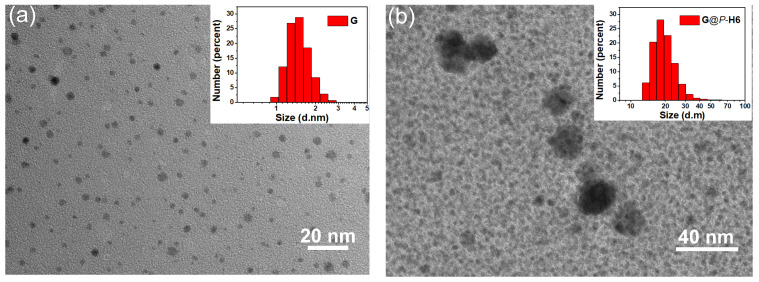
TEM images of (**a**) the silver cluster **G** (inset: DLS image of **G**) and (**b**) the nanocluster complex **G**@*P*-**H6** (inset: DLS image of **G**@*P*-**H6**).

Moreover, the size of **G** would be changed when the surface was covered with the macrocyclic hosts. The TEM images showed the isolated **G** presented particles with good monodispersity and uniform particle size (~2 nm) (Figure 4a). The nanocluster complex **G**@*P*-**H6** with the diameter of 18 nm was much larger than the isolated **G** (Figure 4b), which was attributed to the cap of *P*-**H6** on the surface of the silver cluster. It is worth noting that the size of **G**@*P*-**H6** was much larger than the length of one **G** and two *P*-**H6** molecules. We speculated that further aggregation through the π–π interaction afforded the increasing size of the nanocluster complexes because the isolated hosts presented aggregated particles (~12 nm) in TEM images (Figure S11). Besides, the average hydrodynamic diameters of **G** and **G**@*P*-**H6** measured by DLS were in agreement with the TEM results. The silver cluster **G** possessed an average hydrodynamic diameter of 1.4 nm in solution (Figure 4a, inset). For complex **G**@*P*-**H6**, the average size was about 20 nm (Figure 4b, inset). The nanocluster complex formed by *M*-enantiomer showed the same morphological results (Figures S12 and S13). These results not only proved the formation of the nanocluster complexes, but also suggested that the macrocyclic hosts had a dramatic influence on the size and surface environment of the silver cluster.

### 2.3. DFT Calculation of the Nanocluster Complexes

**Figure 5:**
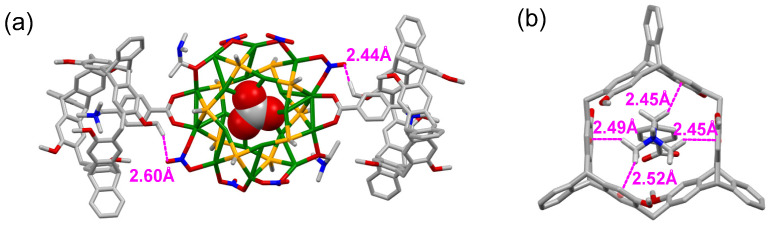
(**a**) DFT optimized structure of complex **G**@*P*-**H6** and the hydrogen-bonding distances between **G** and *P*-**H6**. (**b**) The C−H···π interaction distances between the **L1-H** ligand of **G** and *P*-**H6**. H atoms not involved in the noncovalent interactions were omitted for clarity. Color legend: Ag, green; S, orange; C, gray; N, blue; O, red.

In addition, DFT calculation was further implemented at the PBE [36]/(DND:DSPP) [37] level of theory by the Dmol^3^ package [38,39] to shed light on the structure of the inclusion complex **G**@*P*-**H6**. In the theoretical model of **G** and **G**@*P*-**H6**, the ligand **L2** was simplified as SCH_3_, and the two NO_3_^−^ anions that covered the top and the bottom of the Ag_20_S_10_ core were omitted. The optimized structure of **G** (Figure S22) was consistent with the single-crystal structure. As shown in Figure 5a,b, two *P*-**H6** hosts encapsulated the two **L1-H** ligands of **G**, respectively. It was revealed that C−H∙∙∙π and hydrogen-bonding interactions were responsible for such complexation. When the **L1-H** ligand of **G** was included in the cavity of *P*-**H6**, the C−H···π interaction distances were 2.45 to 2.52 Å and the hydrogen-bonding distances of 2.44 to 2.60 Å were found between O of the NO_3_^−^ ligands and the nearest H of *P*-**H6**. The binding energy (BE) value in the PBE method for **G**@*P*-**H6** was −282.81 kcal/mol. All the simulated results confirmed the stable complexation between the silver cluster **G** and the host *P*-**H6**.

### 2.4. Photophysical Properties of the Nanocluster Complexes

**Figure 6:**
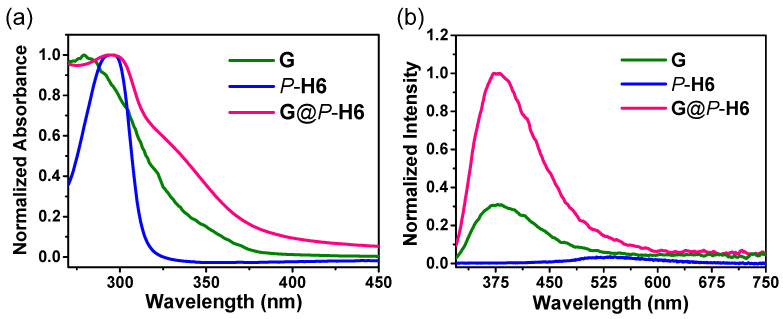
(**a**) Absorption and (**b**) photoluminescence spectra of **G**, *P*-**H6**, and **G**@*P*-**H6** in solid state at 300 K (λ_ex_ = 290 nm).

The absorption spectra of the silver cluster, macrocyclic hosts, and their complexes were then investigated both in solution and solid state. It could be observed that the UV-Vis absorption spectra of *P*-**H6** showed a maximum absorption peak at 290 nm, and **G** presented a broad absorption band from 267 to 350 nm in solution (Figure S14). When *P*-**H6** and **G** were dissolved in solution, the complex showed absorption at 290 nm with a shoulder peak at about 325 nm (Figure S14). As shown in Figure 6a, the UV-Vis absorption spectra in solid state were basically consistent with those in solution. **G** and *P*-**H6** displayed a maximum absorption peak at 280 and 290 nm, respectively. The absorption spectrum of the nanocluster complexes exhibited a broad absorption peak from 275 to 375 nm with a new shoulder peak at ca. 340 nm due to the host−guest complexation between *P*-**H6** and the ligand of **G**. Solid-state absorption spectra of **G** with different equivalents of *P*-**H6** were also measured (Figure S16) to further investigate the host–guest complexation. It could be found that the increase of *P*-**H6** caused a bathochromic shift of the maximum absorption peak, and the new shoulder peak at 325 nm gradually increased. These results might be attributed to the overlap of the absorption peaks and the host−guest complexation of *P*-**H6** and **G**.

The solid-state photoluminescence properties of **G**, *P*/*M*-**H6**, and **G**@*P*/*M*-**H6** at 300 and 77 K were further studied. As shown in Figure 6b, **G** showed a weak emission band centered at 370 nm at 300 K and the luminescence of *P*-**H6** was barely detected under the same test conditions. With the increase of *P*-**H6**, the emission of **G** at 375 nm gradually enhanced until the molar ratio of *P*-**H6**/**G** reached 2/1 (Figure S18). Compared with that of the free silver cluster, the maximum emission peak of **G**@*P*-**H6** at 375 nm enhanced nearly three times. It was probably because the trimethylammonium of **L1-H** was encapsulated into the cavity of *P*-**H6** so that the rigidity of **G** enhanced and its non-radiative transition was suppressed.

**Figure 7:**
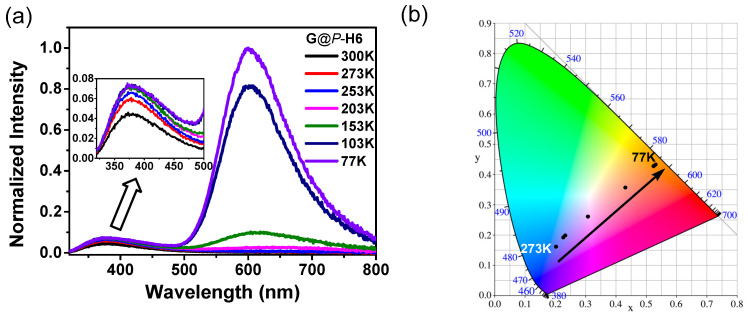
(**a**) Solid-state emission spectrum of **G**@*P*-**H6** at different temperatures and (**b**) CIE chromaticity diagram showing the color coordinates of **G**@*P*-**H6** according to the temperature-dependent emission spectrum.

Moreover, we also measured the temperature-dependent luminescence of **G** and **G**@*P*/*M*-**H6** (Figure 7a, Figures S19 and S20). It was found when the temperature of **G**@*P*-**H6** decreased from 300 to 77 K, a distinct emission at 600 nm appeared and gradually enhanced. The maintained low-temperature emission at 600 nm of **G**@*P*-**H6** could be assigned to the cluster-centered excited state and ligand-to-metal charge-transfer transition [34,40]. However, the luminescence intensity at 77 K of **G**@*P*-**H6** at 600 nm was obviously lower than that of the isolated **G** (Figure S21). These observations could be attributed to the protection to the singlet excited state of **G** from the cavity of *P*-**H6**, which effectively reduced the loss of energy by non-radiative decay at 300 K and decelerated the intersystem crossing process of **G** at 77 K. Besides, the temperature-dependent spectrum of **G**@*P*-**H6** was transformed to the Commission Internationale de L’Eclairage (CIE) 1931 coordinates (Figure 7b). It could be observed that the emission colors of **G**@*P*-**H6** changed from orange to purplish blue with the increase of temperature in the CIE chromaticity diagram, which presented multicolor luminescence with a broad range.

**Figure 8:**
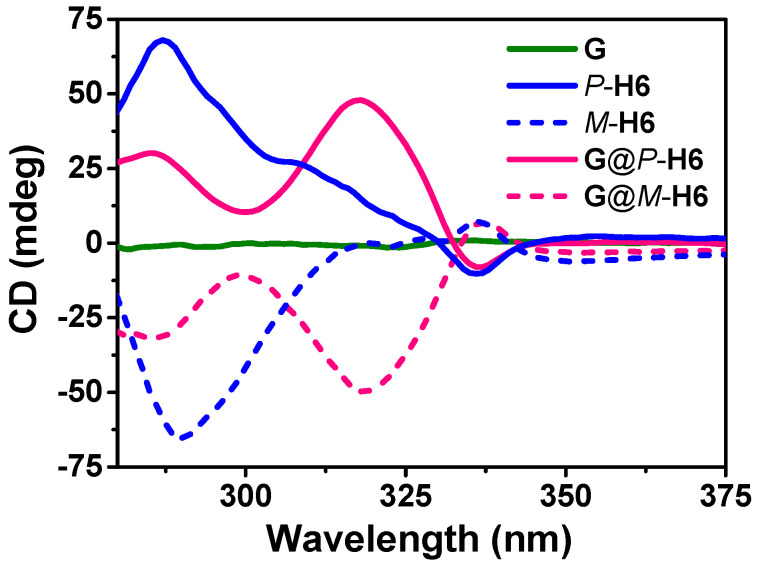
The CD spectra of *P*/*M*-**H6** hosts, silver cluster **G**, and nanocluster complexes **G**@*P*/*M*-**H6** in the solid state.

Based on the spatial confinement of the silver clusters by the chiral cavities of the macrocyclic hosts, we deduced that the nanocluster complexes could show chiroptical properties in the solid state. As shown in Figure 8, mirrored Cotton effects for *P*-**H6** and *M*-**H6** from 275 to 325 nm were obviously observed, in agreement with their absorption regions, while the silver cluster **G** exhibited no CD signals. The complexes of **G**@*P*-**H6** and **G**@*M*-**H6** presented mirrored CD signals from 275 to 340 nm with a new maximum peak at 320 nm, which suggested that the achiral silver cluster was endowed with CD properties. Since the Ag_20_S_10_ core and ligands of **G** were achiral, the new CD responses might arise from a tandem process: chiral cavities of *P*/*M*-**H6** induced chiral character in the electronic transitions of ligand **L1-H**, and then ligand-to-silver core-based electronic transitions led to the CD activity. However, CPL signals of **G**@*P*/*M*-**H6** were not detected, which might be caused by the reduced efficiency of chirality transfer during the two transition processes. These results indicated that the host−guest assembly with chiral macrocyclic hosts could be a convenient and potent method to endow achiral metal nanoclusters with chiroptical properties.

## 3. Conclusions

In summary, a pair of new chiral nanocluster complexes based on the enantiomeric 2,6-helic[6]arenes and achiral Ag_20_ clusters were conveniently prepared and characterized experimentally and theoretically. The emission spectra showed that the nanocluster complexes possessed enhanced luminescence at room temperature due to the protection to the singlet excited state of the silver clusters from the encapsulation of the host cavities. With the decrease of temperature, the nanocluster complexes also displayed multicolor luminescence with a broad range. More importantly, the complexation between the chiral macrocyclic hosts and the silver clusters endowed the nanocluster complexes with induced CD properties, which suggested the consecutive chirality transfer from the chiral macrocycles to the achiral silver clusters. This work can provide guidance for the design and construction of new functional nanomaterials with chiroptical activity.

## 4. Materials and Methods

### 4.1. General Methods

All reagents and solvents were purchased from commercial sources and used without further purification. ^1^H, ^13^C, and 2D NOESY NMR spectra were recorded on Brucker^®^ AVIII 400, 600, and 700 MHz NMR spectrometers. Single-crystal data were collected on XtaLAB Synergy-R using graphite mono-chromated Cu Kα radiation. UV-Vis spectra were recorded on a PerkinElmer^®^ UV/Vis/NIR spectrometer (Lambda 950), and the fluorescence spectra were recorded on an Edinburgh Instruments FLS1000 spectrometer. CD spectra were recorded on a JASCO J815 spectropolarimeter. TEM images were obtained on a Hitachi^®^ HT-7700. DLS measurements were implemented on Zetasizer Nano ZS ZEN3600 of Malvern Instruments Ltd. The hosts *P*-**H6**/*M*-**H6**, trimethylammonium **L1** ligands, and precursor [AgS*t*Bu]_n_ were prepared according to the literature procedure [24,41,42]. Normalization of the photoluminescence spectra was performed by dividing the luminescence intensities by the maximum emission of the host–guest complex at 375 nm.

### 4.2. Synthetic Procedure of the Silver Cluster and the Nanocluster Complexes

**G**: To an 8 mL mixed solvent of acetonitrile and N,N-dimethylacetamide (*v*/*v* = 1:1), Ag_20_ precursor [AgS*t*Bu]_n_ (0.1 mmol, 19.7 mg) was added under vigorous stirring at room temperature. Then, AgNO_3_ (0.2 mmol, 34 mg) and the **L1** ligand (0.03 mmol, 10.2 mg) were added successively. When **L1** was completely dissolved, 40 μL of triethylamine was added. The mixture was allowed to react for another 30 min. Finally, the resultant solution was filtered with syringe filters and injected into a test tube. With the poor solvent tetrahydrofuran diffusing into the filtrate, light-brown block crystals of **G** were obtained in darkness after three days. Yield: 65% (based on Ag). Elemental analysis calcd. (%) for C_71_H_138_N_12_S_10_Ag_20_O_33_ (4144.77): C 20.47; H 3.34; N 4.03; S 7.70; found: C 20.48; H 3.37; N 4.07; S 7.66.

**G**@*P*-**H6**: The host *P*-**H6** (14.2 mg, 0.015 mmol) and the crystals of **G** (31 mg, 0.0075 mmol) were dissolved in 6 mL of mixed solvent of CH_3_CN and DMSO (*v*/*v* = 5:1). After stirring for 30 min, ether was added to precipitate complex **G**@*P*-**H6**. The turbid liquid was centrifuged, and its supernatant was discarded. Then, the white nanocluster complex was collected after the residual solvent was evaporated. The enantiomeric complex **G**@*M*-**H6** was synthesized by the same method.

### 4.3. Preparation of the Samples for CD Measurements

The solid-state samples for CD measurements were ground with KBr (mass ratio of *P*-**H6**/KBr = 1:77, mass ratio of **G**/KBr = 2:77) and then compressed to transparent discs.

## Data Availability

The data presented in this study are openly available in the article and Supplementary Materials.

## Supplementary Materials

The following supporting information can be downloaded at: https://www.mdpi.com/article/10.3390/molecules27123932/s1: Figure S1: (a) ^1^H NMR spectra (400 MHz, CD_3_CN:DMSO-*d_6_* = 5:1 (*v*/*v*), 298 K) of *P***-H6** with different equivalents of **L1**. [*P***-H6**] = 2.50 mM; (b) Plots of Δδ (ppm) for the bridgehead proton vs the concentration of **L1**. Figure S2: Mole ratio of *P***-H6** vs **L1**. Figure S3: (a) ^1^H NMR spectra (400 MHz, CD_3_CN:DMSO-*d_6_* = 5:1 (*v*/*v*), 298 K) of *M***-H6** with different equivalents of **L1**. [*M***-H6**] = 2.50 mM; (b) Plots of Δδ (ppm) for the bridgehead proton vs the concentration of **L1**. Figure S4: Mole ratio of *M***-H6** vs **L1**. Figure S5: ^1^H NMR spectra (400 MHz, CD_3_CN:DMSO-*d_6_* = 5:1 (*v*/*v*), 298 K) of **G** with different equivalents of *P***-H6**. [**G**] = 2.50 mM. Figure S6: (a) The ^1^H NMR spectra of *M*-**H6**, **G** and **G**@*M*-**H6** (400 MHz, CD_3_CN:DMSO-*d_6_* = 5:1 (*v*/*v*), 298 K, [*M*-**H6**] = 2.5 mM, [**G**] = 1.25 mM). Figure S7: 2D NOESY NMR spectrum (700 MHz, CD_3_CN/DMSO-*d_6_* = 5:1 (*v*/*v*), 298 K) of **G**@*P*-**H6**. Figure S8: 2D NOESY NMR spectrum (700 MHz, CD_3_CN/DMSO-*d_6_* = 5:1 (*v*/*v*), 298 K) of **G**@*M*-**H6**. Figure S9: Solid-state ^13^C NMR spectra of *P*-**H6**, **G** and **G**@*P*-**H6** (150 MHz, 298 K, mole ratio of *P*-**H6**/**G** is 2/1). Figure S10: Solid-state ^13^C NMR spectra of *M*-**H6**, **G** and **G**@*M*-**H6** (150 MHz, 298 K, mole ratio of *M*-**H6**/**G** is 2/1). Figure S11: TEM images of the macrocyclic hosts: (a) *P*-**H6**, (b) *M*-**H6**. Figure S12: TEM images of the nanocluster complex **G@***M*-**H6** ([*M*-**H6**]/[**G**] = 2/1). Figure S13: The DLS image of **G@***M*-**H6** ([*M*-**H6**]/[**G**] = 2/1). Figure S14: Normalized UV-Vis absorption spectra of *P*-**H6**, **G** and **G**@*P*-**H6** in CH_3_CN:DMSO = 5:1 (*v*/*v*) ([*P*-**H6**] = 0.02 mM, [**G**] = 0.01 mM). Figure S15: Normalized UV-Vis absorption spectra of *M*-**H6**, **G** and **G**@*M*-**H6** in solid state. Figure S16: Normalized UV-Vis absorption spectra of **G** with different equivalents of *P*-**H6** in solid state. Figure S17: Normalized luminescence spectra of *M*-**H6**, **G** and **G**@*M*-**H6** at 300 K in solid state. Figure S18: Normalized luminescence spectra of **G** with different equivalents of *P*-**H6** in solid state at 300 K. Figure S19: Solid-state emission spectra of **G**@*M*-**H6** at different temperatures. Figure S20: Solid-state emission spectrum of **G** at different temperatures. Figure S21: Normalized luminescence spectra of (a) *P*-**H6**, **G** and **G**@*P*-**H6**; (b) *M*-**H6**, **G** and **G**@*M*-**H6** at 77 K in solid state. Figure S22: (a) Top view and (b) side view of the optimized structure of **G** (H atoms are omitted for clarity; color legend: Ag, green; S, orange; C, gray; N, blue; O, red). Table S1: Crystal data and structure refinements for **G** (CCDC 2156603). Table S2: DFT calculation result of **G**.

## Sample Availability

Samples of the compounds are available from the authors.
