# Supplementary material for: Chiral Nanocluster Complexes Formed by Host−Guest Interaction between Enantiomeric 2,6-Helic[6]arenes and Silver Cluster Ag20: Emission Enhancement and Chirality Transfer
Source: Molecules. 2022 Jun 19;27(12):3932. doi: 10.3390/molecules27123932 (PMC9230552; doi:10.3390/molecules27123932)
Supplement: Supplementary file 1 [file molecules-27-03932-s001.zip › molecules-1782265-supplementary.pdf]

Electronic Supplementary Materials for

Chiral Nanocluster Complexes Formed by Host−Guest Interaction Between Enantiomeric 2,6-Helic[6]arenes and Silver Cluster Ag_{20}: Emission Enhancement and Chirality Transfer

Yan Guo,[1,2] Ying Han[1,]* and Chuan-Feng Chen[1,2,]*

[1]*Beijing National Laboratory for Molecular Sciences, CAS Key Laboratory of Molecular Recognition and Function, Institute of Chemistry, Chinese Academy of Sciences, Beijing 100190, China.* [2]*University of Chinese Academy of Sciences, Beijing 100049, China.*

E-mail: cchen@iccas.ac.cn; hanying463@iccas.ac.cn

Table of Contents

1. **Crystallographic data of the silver cluster...S2**
2. **Investigation of host-guest interaction between the macrocyclic hosts and the ligand L1...S3**
3. **NMR spectra of the macrocyclic hosts, the silver cluster and the nanocluster complexes...S6**
4. **TEM images of the macrocyclic hosts and the nanocluster complexes................S8**
5. **DLS measurements of the nanocluster complexes..S9**
6. **Photophysical properties of the nanocluster complexes.......................................S9**
7. **DFT calculations of the silver cluster and the nanocluster complexes................S14**
8. **References..S30**

1. Crystallographic data of the silver cluster

Table S1. Crystal data and structure refinements for **G** (CCDC 2156603)

Empirical formula	$C_{52.5}H_{96}Ag_{10}N_7O_{16}S_5$
Formula weight	2320.36
Temperature/K	170.00(10)
Crystal system	triclinic
Space group	P-1
a/Å	15.5279(2)
b/Å	15.8626(2)
c/Å	16.8428(2)
α/°	99.7940(10)
β/°	98.5320(10)
γ/°	90.7130(10)
Volume/$Å^3$	4039.87(9)
Z	2
$\rho_{calc}g/cm^3$	1.908
μ/mm^{-1}	20.679
F(000)	2276
Crystal size/mm^3	0.25 × 0.2 × 0.1
Radiation	CuKα (λ = 1.54184)
2Θ range for data collection/°	5.388 to 151.024
Index ranges	-19≤h≤17, -19≤k≤19, -20≤l≤20
Reflections collected	54485
Independent reflections	16027 [R_{int} = 0.0693, R_{sigma} = 0.0553]
Data/restraints/parameters	16027/66/867
Goodness-of-fit on F^2	1.08
Final R indexes [I>=2σ (I)]	R_1 = 0.0595, wR_2 = 0.1614
Final R indexes [all data]	R_1 = 0.0704, wR_2 = 0.1692
Largest diff. peak/hole / e $Å^{-3}$	3.10/-2.99

2. Investigation of host-guest interaction between the macrocyclic hosts and the ligand L1

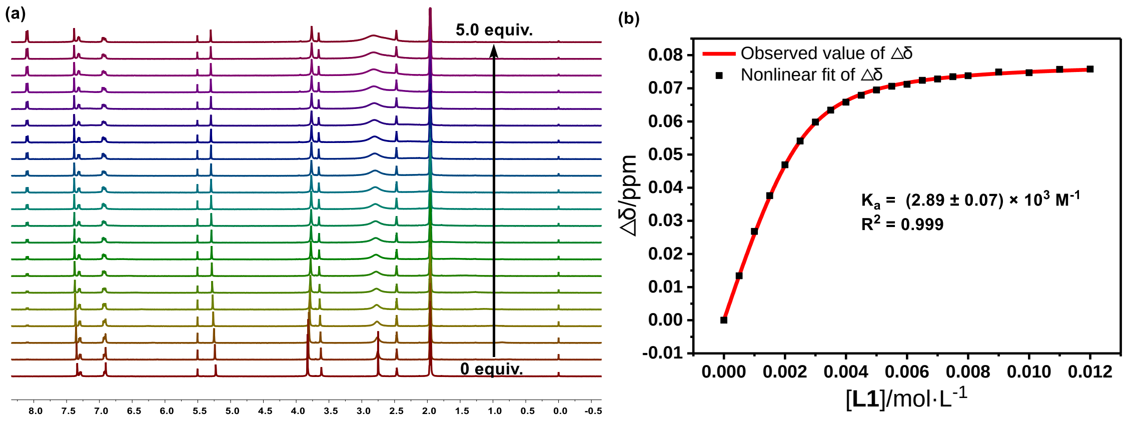

Figure S1. (a) ^{1}H NMR spectra (400 MHz, CD_3CN:DMSO-d_6 = 5:1 (*v*/*v*), 298 K) of ***P*-H6** with different equivalents of **L1**. [***P*-H6**] = 2.50 mM; (b) Plots of Δδ (ppm) for the bridgehead proton vs the concentration of **L1**.

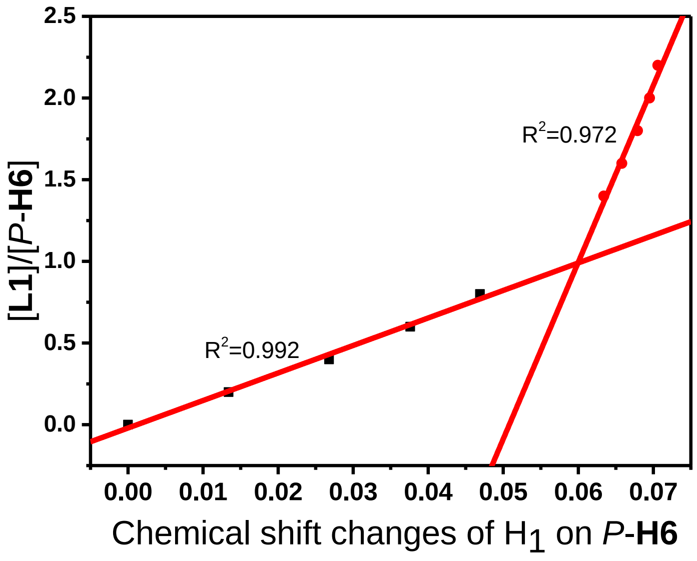

Figure S2. Mole ratio of ***P*-H6** vs **L1**.

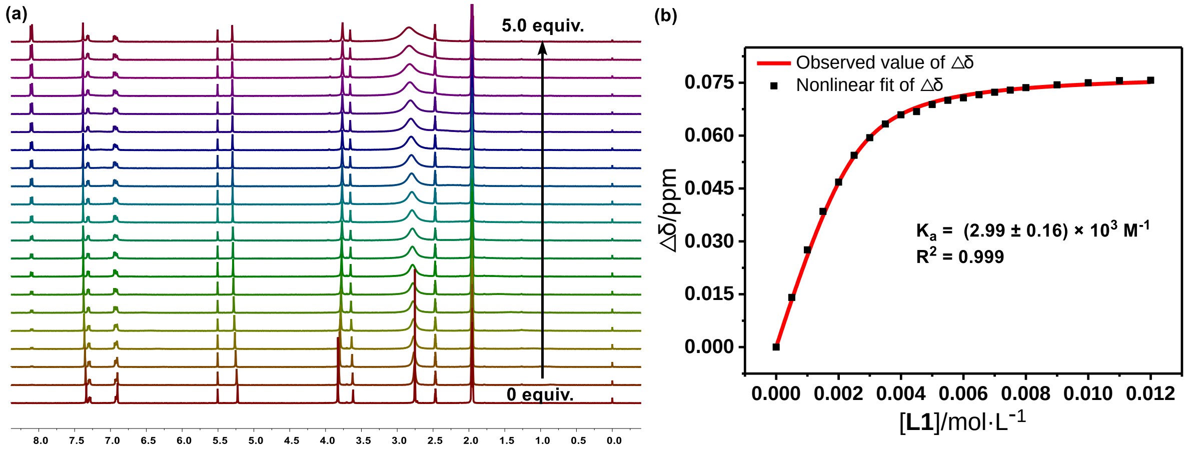

Figure S3. (a) ^{1}H NMR spectra (400 MHz, CD_3CN:DMSO-d_6 = 5:1 (*v*/*v*), 298 K) of *M*-**H6** with different equivalents of **L1**. [*M*-**H6**] = 2.50 mM; (b) Plots of Δδ (ppm) for the bridgehead proton vs the concentration of **L1**.

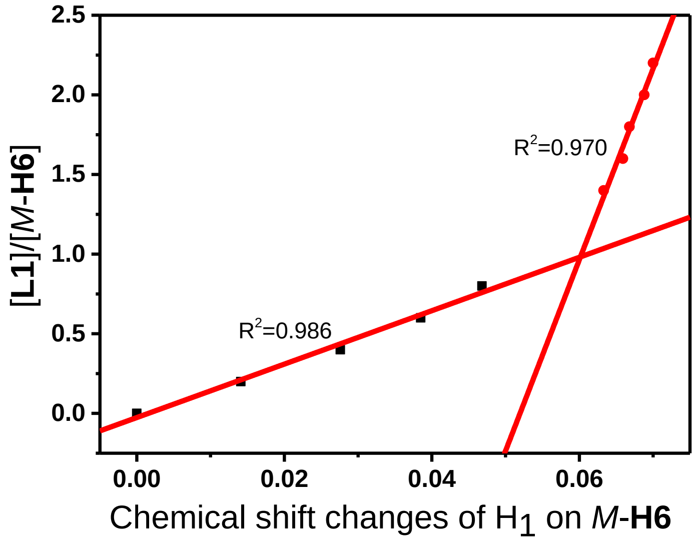

Figure S4. Mole ratio of *M*-**H6** vs **L1**.

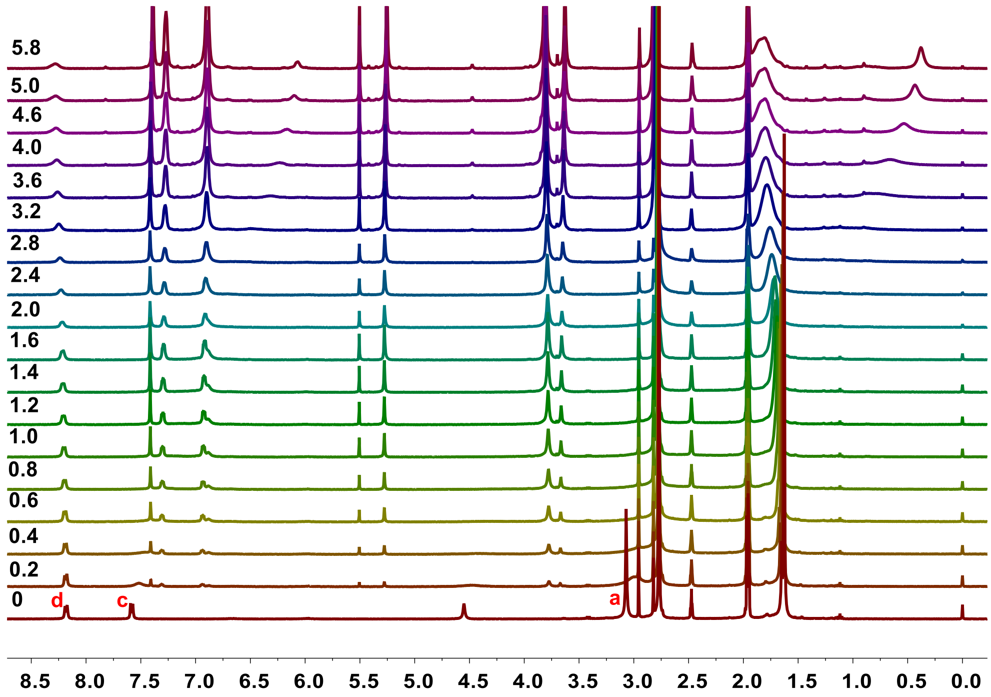

Figure S5. ^{1}H NMR spectra (400 MHz, CD_3CN:DMSO-d_6 = 5:1 (v/v), 298 K) of **G** with different equivalents of *P*-**H6**. [**G**] = 2.50 mM.

3. NMR spectra of the macrocyclic hosts, the silver cluster and the nanocluster complexes

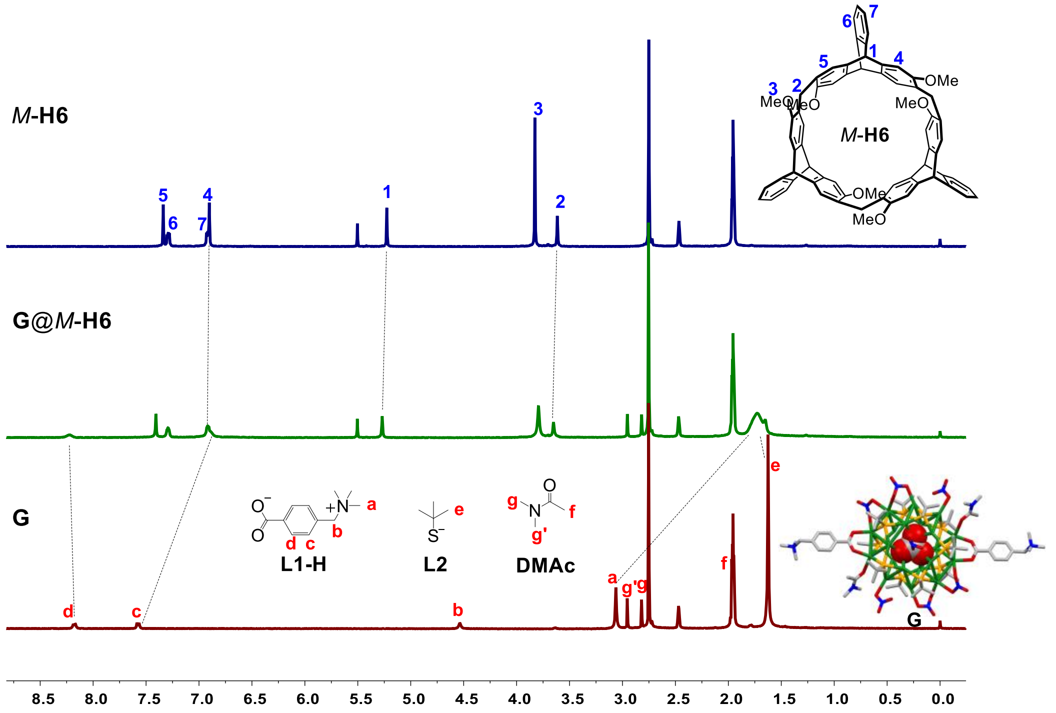

Figure S6. (a) The ^{1}H NMR spectra of *M*-**H6**, **G** and **G@*M*-H6** (400 MHz, CD_3CN:DMSO-d_6 = 5:1 (*v*/*v*), 298 K, [*M*-**H6**] = 2.5 mM, [**G**] = 1.25 mM).

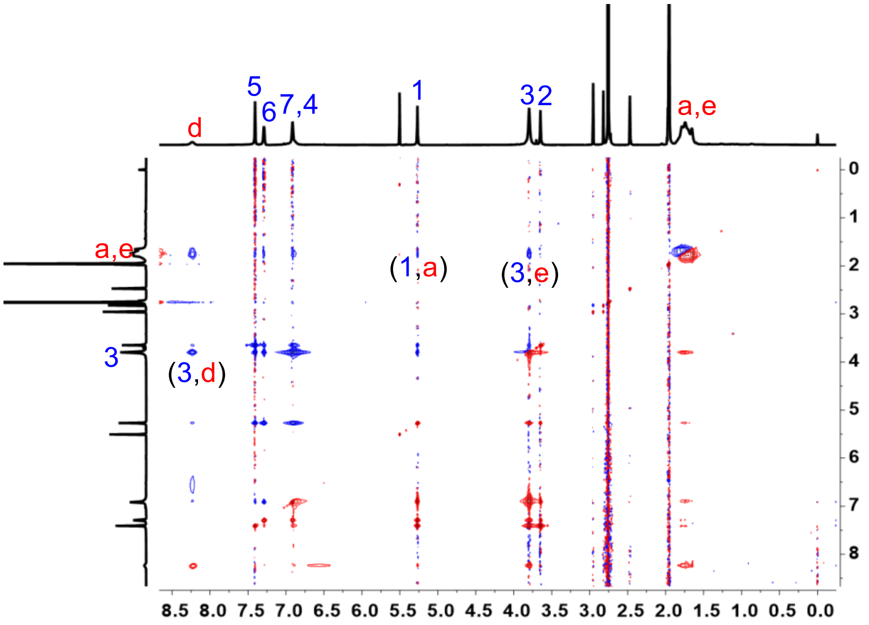

Figure S7. 2D NOESY NMR spectrum (700 MHz, CD_3CN/DMSO-d_6 = 5:1 (*v*/*v*), 298 K) of **G@*P*-H6**.

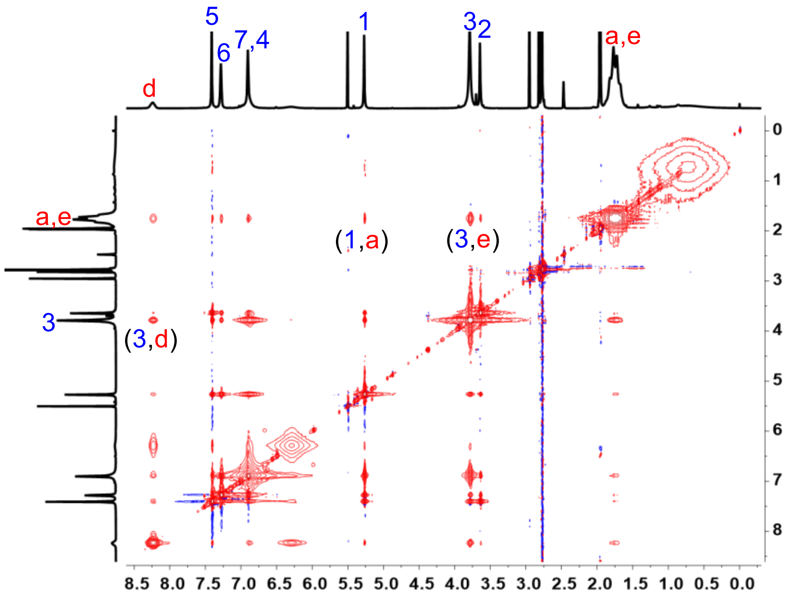

Figure S8. 2D NOESY NMR spectrum (700 MHz, CD_3CN/DMSO-d_6 = 5:1 (v/v), 298 K) of **G@*M*-H6**.

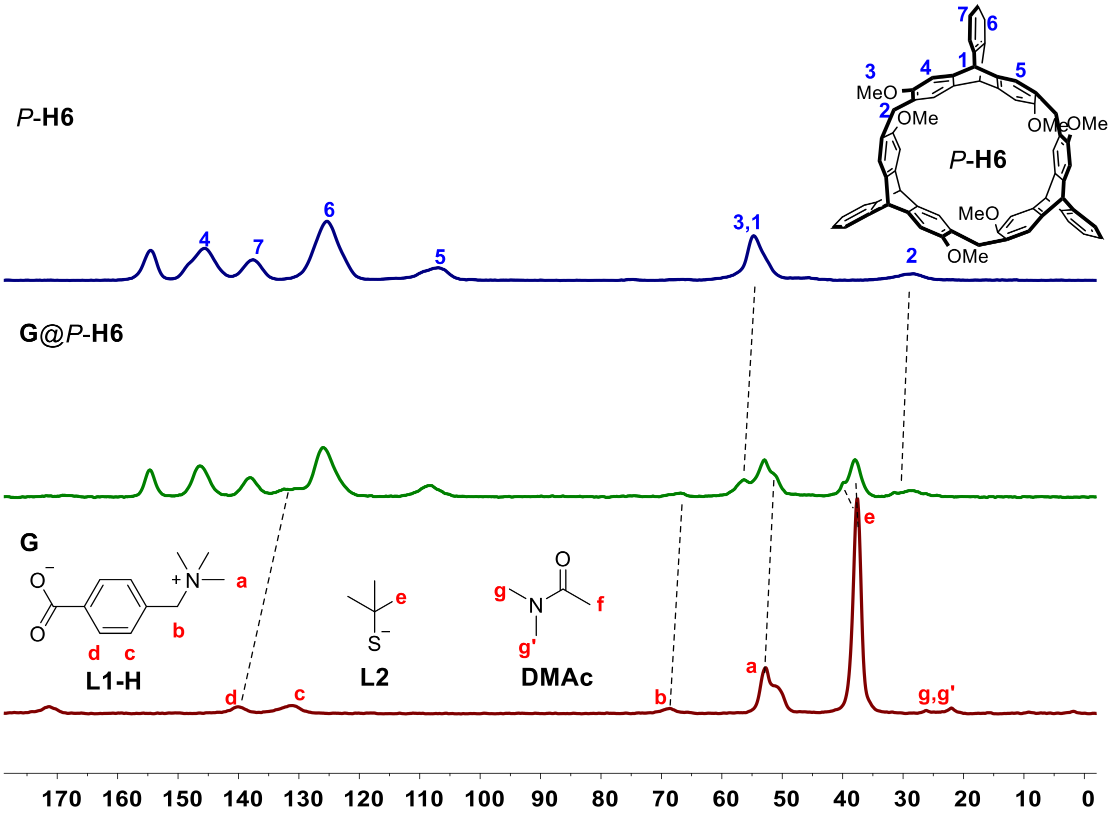

Figure S9. Solid-state ^{13}C NMR spectra of ***P*-H6**, **G** and **G@*P*-H6** (150 MHz, 298 K, mole ratio of ***P*-H6**/**G** is 2/1).

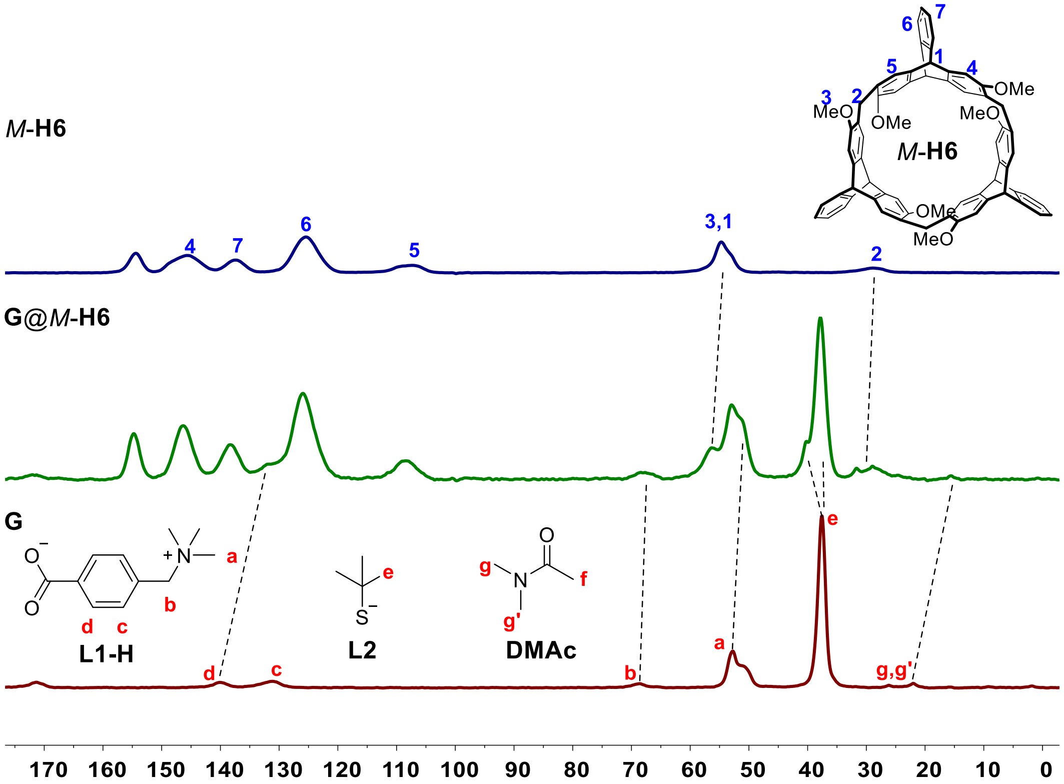

Figure S10. Solid-state ^{13}C NMR spectra of *M*-**H6**, **G** and **G@*M*-H6** (150 MHz, 298 K, mole ratio of *M*-**H6**/**G** is 2/1).

4. TEM images of the macrocyclic hosts and the nanocluster complexes

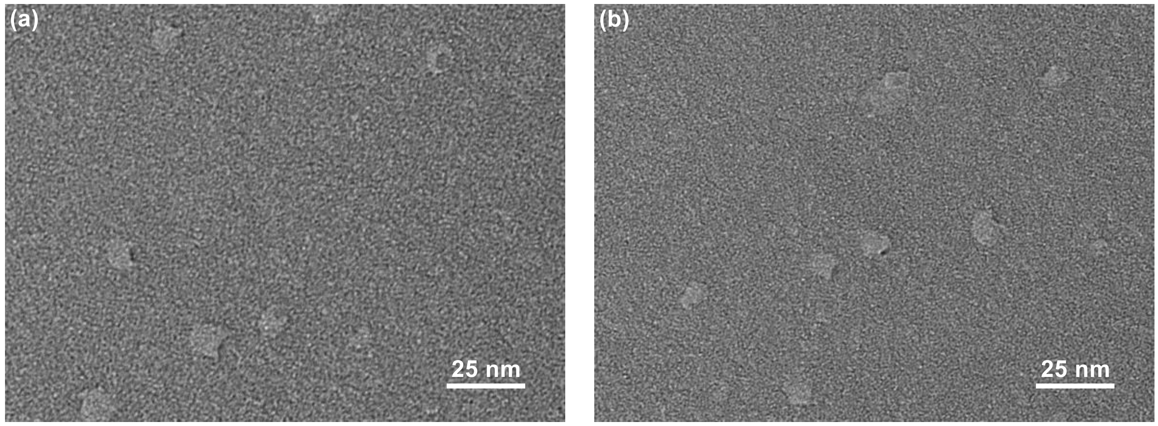

Figure S11. TEM images of the macrocyclic hosts: (a) *P*-**H6**, (b) *M*-**H6**.

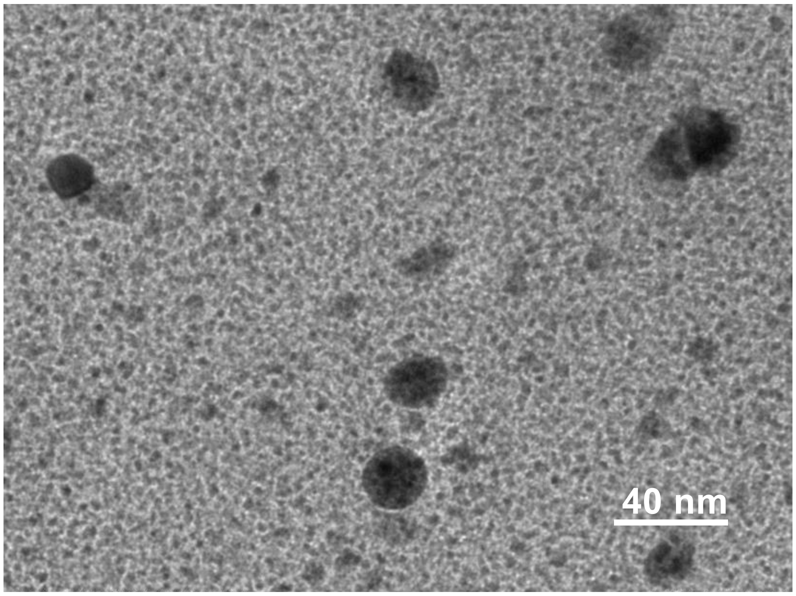

Figure S12. TEM images of the nanocluster complex **G@*M*-H6** ([***M*-H6**]/[**G**] = 2/1).

5. DLS measurements of the nanocluster complexes

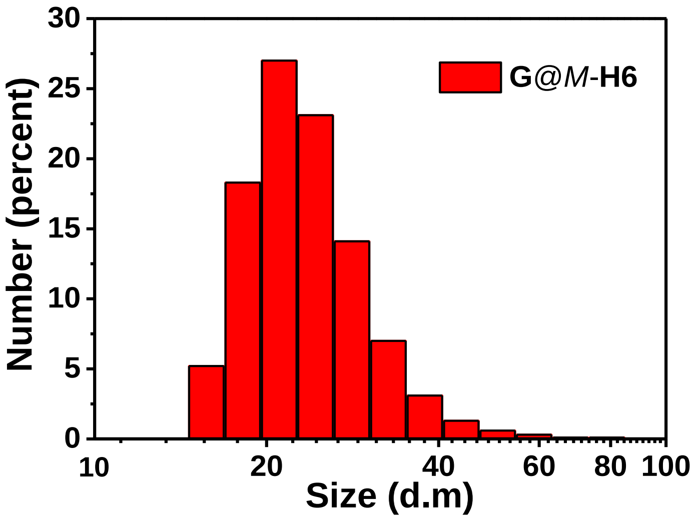

Figure S13. The DLS image of **G@*M*-H6** ([***M*-H6**]/[**G**] = 2/1).

6. Photophysical properties of the nanocluster complexes

The solid-state samples for UV-Vis absorption and CD measurements were ground with KBr (mass ratio of ***P*-H6**/KBr = 1:77, mass ratio of **G**/KBr = 2:77) and then compressed to transparent discs.

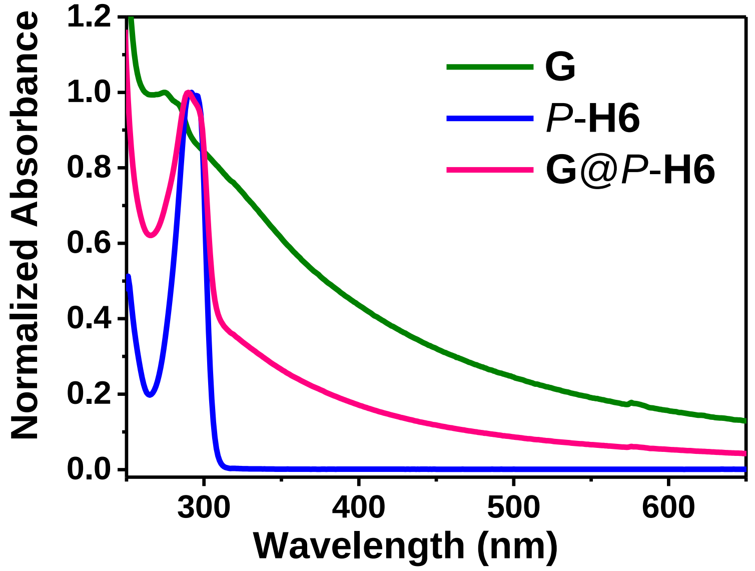

Figure S14. Normalized UV-Vis absorption spectra of *P*-**H6**, **G** and **G@*P*-H6** in CH_3CN:DMSO = 5:1 (*v*/*v*) ([*P*-**H6**] = 0.02 mM, [**G**] = 0.01 mM).

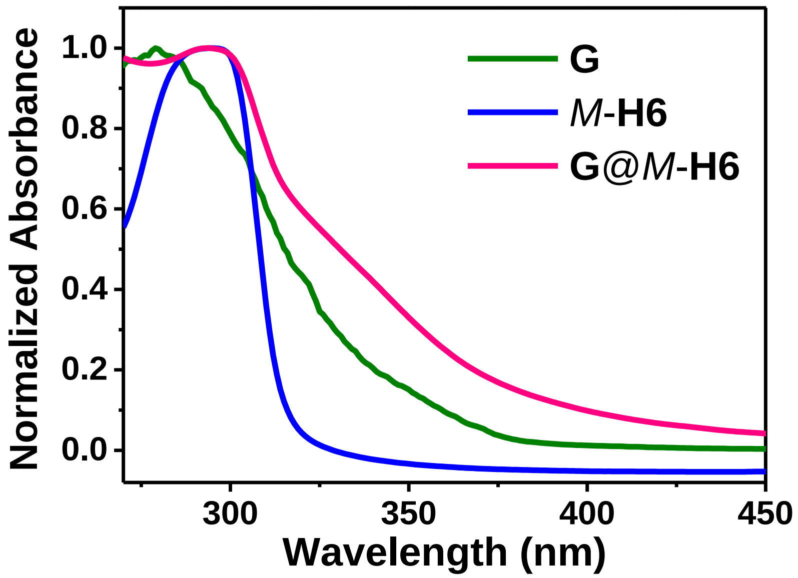

Figure S15. Normalized UV-Vis absorption spectra of *M*-**H6**, **G** and **G@*M*-H6** in solid state.

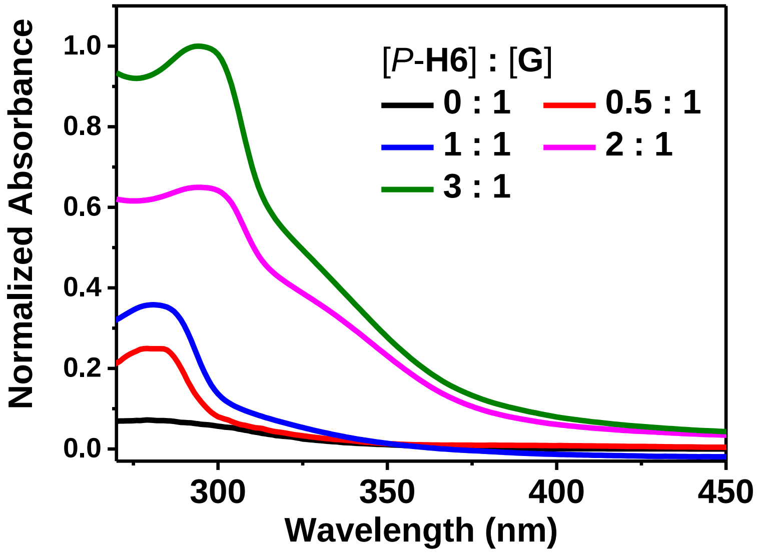

Figure S16. Normalized UV-Vis absorption spectra of **G** with different equivalents of *P*-**H6** in solid state.

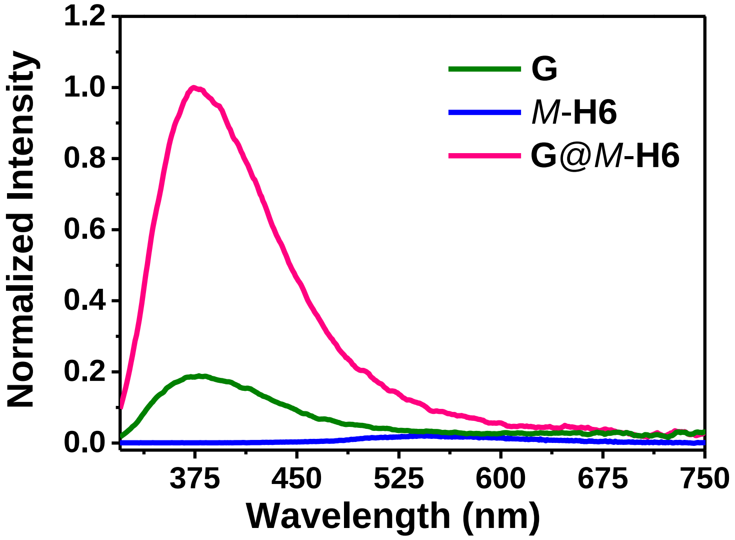

Figure S17. Normalized luminescence spectra of *M*-**H6**, **G** and **G@*M*-H6** at 300 K in solid state.

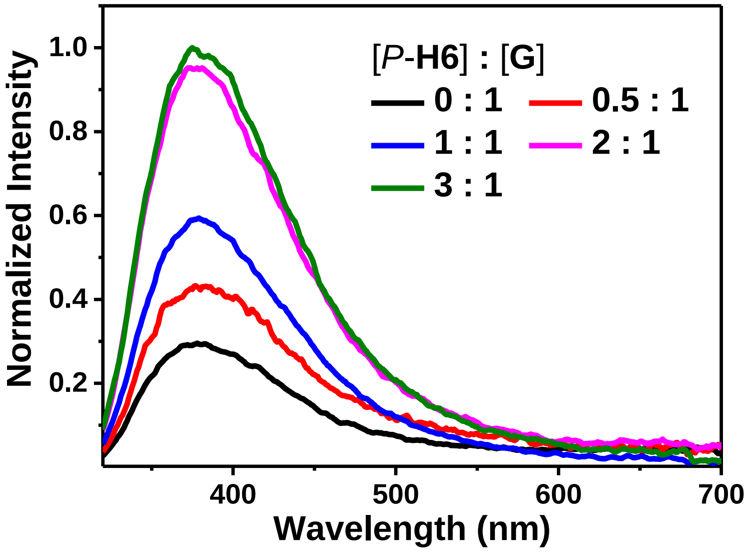

Figure S18. Normalized luminescence spectra of **G** with different equivalents of *P*-**H6** in solid state at 300 K.

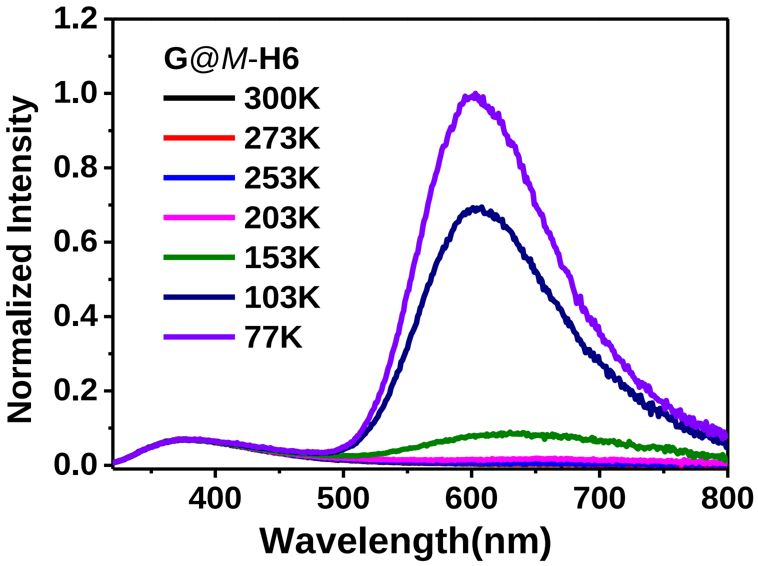

Figure S19. Solid-state emission spectra of **G@***M***-H6** at different temperatures.

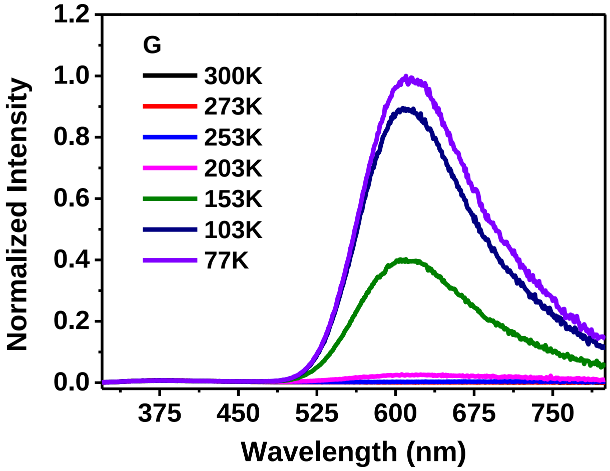

Figure S20. Solid-state emission spectrum of **G** at different temperatures.

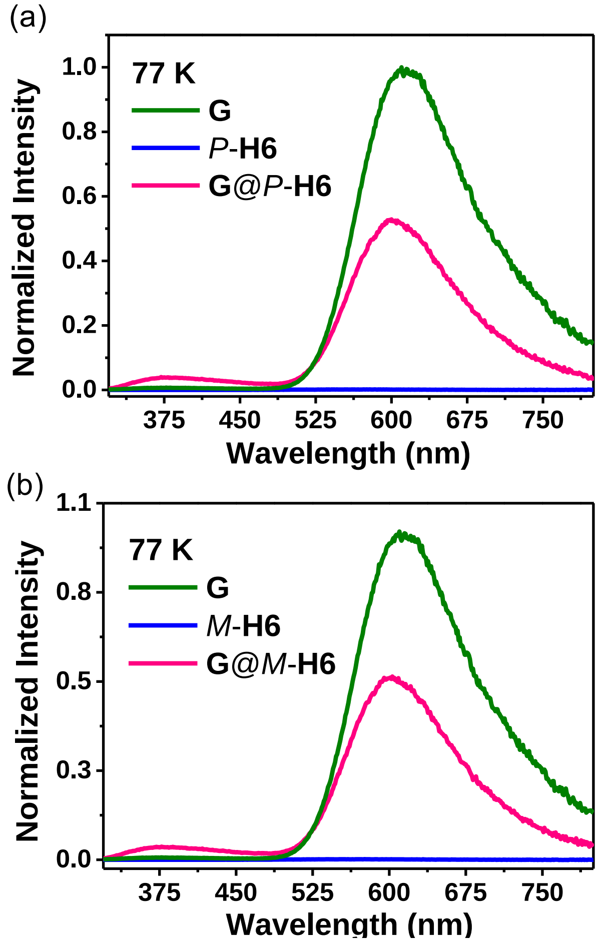

Figure S21. Normalized luminescence spectra of (a) *P*-**H6**, **G** and **G@*P*-H6**; (b) *M*-**H6**, **G** and **G@*M*-H6** at 77 K in solid state.

7. DFT calculations of the silver cluster and the nanocluster complexes

The density functional theory (DFT) was performed with Dmol[3] package.[1,2] The geometry optimization of the modeling **G** and **G@*P*-H6** was carried out with GGA/PBE level of theory.[3] The *d*-polarization included DND basis set was used for C, H, O, N, S and the DFT Semi-core pseudopotial (DSPP) with the associated relativistic corrections was used for Ag.[4]

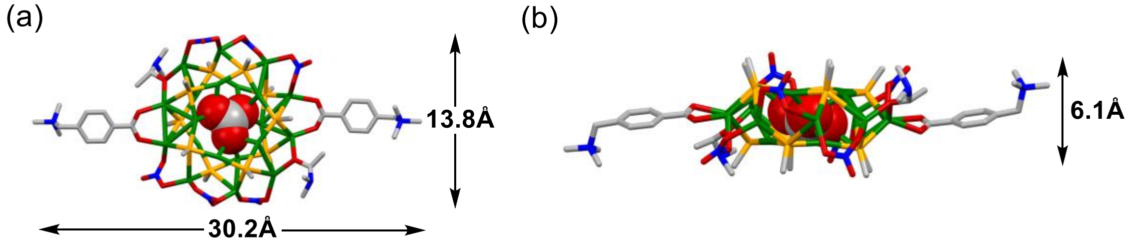

Figure S22. (a) Top view and (b) side view of the optimized structure of **G** (H atoms are omitted for clarity; color legend: Ag, green; S, orange; C, gray; N, blue; O, red).

Table S2. DFT calculation result of **G**.

Ag	-2.53280000	-1.70610000	1.52940000
Ag	1.13290000	-4.76070000	2.03440000
Ag	-0.62470000	1.70950000	-3.20640000
Ag	1.23350000	-1.17040000	-2.94200000
Ag	3.05500000	-4.42560000	-0.65180000
Ag	0.07780000	3.33840000	0.52840000
Ag	-1.42500000	-3.64800000	4.13030000
Ag	4.36570000	-2.26450000	-2.87490000
Ag	-2.94850000	1.36800000	-0.02010000
Ag	3.48690000	1.07980000	-4.55700000
S	-1.60100000	-3.93770000	1.46890000
S	-2.37890000	2.72180000	-1.90850000
S	1.42900000	-3.51860000	-2.22420000
S	-4.05850000	0.18990000	1.76120000
S	1.06850000	0.63770000	-4.54150000
O	6.13930000	-3.48840000	-2.02880000
O	3.75470000	-6.36430000	0.26490000

O	1.84580000	-6.86440000	1.43630000
O	5.23630000	-3.46950000	0.00660000
N	6.25630000	-3.65600000	-0.75330000
O	0.64410000	-4.48890000	4.29020000
O	7.35130000	-3.99370000	-0.27190000
O	4.40780000	-0.96830000	-5.47520000
O	-3.67040000	-3.90960000	4.78520000
C	3.62390000	-10.33250000	2.92950000
H	3.10090000	-10.96590000	3.65840000
C	4.91150000	-10.69350000	2.48310000
O	3.35920000	-2.83190000	-4.91690000
N	3.58310000	-1.90240000	-5.78410000
C	5.56000000	-9.89060000	1.52280000
H	6.54700000	-10.18820000	1.14750000
N	6.46400000	-11.56530000	4.32640000
O	-4.94890000	-2.42030000	3.74450000
N	0.60670000	-5.29850000	6.56890000
C	3.01180000	-9.17840000	2.44200000
H	2.01580000	-8.87430000	2.77430000
C	3.67190000	-8.36510000	1.50440000
C	4.94700000	-8.73550000	1.04100000
H	5.43630000	-8.09100000	0.30390000
C	0.07950000	0.04640000	0.00250000
C	-3.90580000	2.94930000	-2.89260000
N	-4.63620000	-3.65220000	3.97130000
O	-5.26860000	-4.56480000	3.41480000
C	3.02730000	-7.08230000	1.01860000
C	5.59760000	-11.87890000	3.04640000
H	6.32620000	-12.32130000	2.34600000
H	4.89360000	-12.65480000	3.39080000
O	3.00470000	-1.92680000	-6.88700000
C	0.41980000	0.03450000	-6.14610000
C	-5.68980000	-0.34230000	1.11150000
C	7.53010000	-10.56640000	3.99260000
H	8.14900000	-10.97050000	3.17740000
H	8.14200000	-10.39630000	4.89100000
H	7.04720000	-9.63410000	3.66650000
C	-2.89060000	-5.12970000	0.92980000
C	0.85350000	-5.59110000	5.13910000
C	1.05710000	-4.74080000	-3.53030000
C	5.59480000	-11.01500000	5.41560000

H	5.13100000	-10.08530000	5.05580000
H	6.22210000	-10.82240000	6.29910000
H	4.81640000	-11.75570000	5.65210000
C	7.09530000	-12.84460000	4.77890000
H	6.29760000	-13.56460000	5.01680000
H	7.71090000	-12.65040000	5.67000000
H	7.72160000	-13.23200000	3.96080000
C	2.27880000	-6.12240000	4.94450000
H	2.48870000	-6.94820000	5.65060000
H	3.01620000	-5.31570000	5.10200000
H	2.38920000	-6.48650000	3.90970000
C	1.33480000	-4.13410000	7.05850000
H	2.41430000	-4.24700000	6.86550000
H	1.18360000	-4.04870000	8.15040000
H	0.99050000	-3.18970000	6.57850000
C	-0.80590000	-5.17010000	6.89300000
H	-1.25260000	-4.19670000	6.56350000
H	-0.94560000	-5.23130000	7.98940000
H	-1.37750000	-5.98470000	6.41620000
O	0.25770000	1.21230000	-0.54950000
O	0.57600000	-1.01270000	-0.57220000
O	-0.58250000	-0.06480000	1.12070000
Ag	2.48780000	1.80700000	-1.50220000
Ag	-0.98030000	4.93300000	-2.06730000
Ag	0.66610000	-1.48570000	2.87810000
Ag	-1.22130000	1.43670000	3.13890000
Ag	-3.01050000	4.58000000	0.51240000
Ag	-0.16470000	-3.31590000	-0.37200000
Ag	1.79730000	3.88700000	-4.03620000
Ag	-4.37080000	2.46990000	2.88720000
Ag	3.20060000	-1.19150000	-0.00020000
Ag	-3.38790000	-0.92240000	4.53980000
S	1.52930000	4.07020000	-1.34420000
S	2.49770000	-2.59210000	1.81590000
S	-1.56000000	3.67080000	2.27580000
S	4.17320000	0.07690000	-1.80030000
S	-0.92890000	-0.57570000	4.47550000
O	-6.10980000	3.80610000	2.11010000
O	-3.77900000	6.39680000	-0.51320000
O	-1.90070000	7.00320000	-1.68290000
O	-5.23790000	3.48830000	0.09110000

N	-6.19070000	3.94110000	0.82730000
O	-0.30640000	4.48650000	-4.25470000
O	-7.17980000	4.49860000	0.32220000
O	-4.31980000	1.07250000	5.45500000
O	3.99550000	4.23800000	-4.43170000
C	-3.74130000	10.55670000	-2.87360000
H	-3.21170000	11.28290000	-3.50470000
C	-5.06020000	10.82890000	-2.45340000
O	-3.39850000	3.02360000	5.00070000
N	-3.47050000	1.98960000	5.76380000
C	-5.71480000	9.90710000	-1.60890000
H	-6.72930000	10.12680000	-1.25180000
N	-6.54940000	11.79540000	-4.30420000
O	5.21600000	2.44190000	-3.95010000
N	-0.83710000	4.30350000	-6.60650000
C	-3.10830000	9.37740000	-2.48500000
H	-2.08820000	9.14540000	-2.80320000
C	-3.77230000	8.44950000	-1.66250000
C	-5.07790000	8.72970000	-1.21880000
H	-5.57110000	7.99910000	-0.56850000
C	3.86950000	-2.87660000	2.99080000
N	4.94230000	3.68530000	-3.75270000
O	5.60500000	4.34390000	-2.93480000
C	-3.08260000	7.16690000	-1.24850000
C	-5.76900000	12.03150000	-2.94570000
H	-6.55500000	12.38170000	-2.25550000
H	-5.08490000	12.86400000	-3.18340000
O	-2.73090000	1.88750000	6.75980000
C	-0.17750000	-0.23000000	6.11000000
C	5.79970000	0.68980000	-1.20990000
C	-7.59620000	10.74150000	-4.11400000
H	-8.29260000	11.07520000	-3.33080000
H	-8.12820000	10.60140000	-5.06700000
H	-7.10160000	9.80980000	-3.80340000
C	2.80590000	5.29760000	-0.86380000
C	-0.73610000	5.16660000	-5.40820000
C	-1.08560000	4.91950000	3.52820000
C	-5.59860000	11.35820000	-5.37570000
H	-5.12560000	10.41730000	-5.06040000
H	-6.16530000	11.21720000	-6.30830000
H	-4.83370000	12.13820000	-5.50650000

C	-7.19480000	13.08500000	-4.70120000
H	-6.40950000	13.84390000	-4.83740000
H	-7.74480000	12.93590000	-5.64250000
H	-7.88490000	13.39580000	-3.90260000
C	-2.06560000	5.87360000	-5.13440000
H	-2.44710000	6.35810000	-6.05290000
H	-2.81530000	5.15320000	-4.76800000
H	-1.92040000	6.62790000	-4.34190000
C	-1.75820000	3.18370000	-6.46210000
H	-2.77570000	3.54440000	-6.23660000
H	-1.79640000	2.62660000	-7.41740000
H	-1.45100000	2.47490000	-5.65690000
C	0.46610000	3.83060000	-7.05730000
H	0.90730000	3.04670000	-6.39680000
H	0.37620000	3.40170000	-8.07410000
H	1.17470000	4.67780000	-7.09510000
H	0.14660000	-6.42480000	4.91040000
H	0.00770000	5.94290000	-5.70260000
H	-3.65960000	3.56350000	-3.77080000
H	-4.65110000	3.45070000	-2.25460000
H	-4.28670000	1.96920000	-3.21880000
H	0.21530000	0.90920000	-6.78220000
H	1.17840000	-0.61390000	-6.60760000
H	-0.51020000	-0.52560000	-5.97140000
H	-6.29920000	0.55570000	0.93170000
H	-6.15050000	-0.98960000	1.87430000
H	-5.55340000	-0.90260000	0.17570000
H	-2.40470000	-6.10680000	0.78110000
H	-3.65190000	-5.19720000	1.72030000
H	-3.34990000	-4.79400000	-0.01140000
H	1.82370000	-4.64260000	-4.31300000
H	1.06720000	-5.74890000	-3.08960000
H	0.06360000	-4.52710000	-3.95590000
H	3.49170000	-3.52570000	3.79520000
H	4.68670000	-3.36100000	2.43310000
H	4.21040000	-1.91620000	3.40790000
H	0.01350000	-1.19490000	6.60400000
H	-0.88280000	0.38070000	6.69070000
H	0.77040000	0.30850000	5.96490000
H	6.49390000	-0.16420000	-1.18940000
H	6.13570000	1.45390000	-1.92630000

H	5.69980000	1.12040000	-0.20360000
H	2.33110000	6.29130000	-0.83270000
H	3.60870000	5.28760000	-1.61660000
H	3.21760000	5.04870000	0.12570000
H	-1.80860000	4.84250000	4.35450000
H	-1.11410000	5.91510000	3.06080000
H	-0.07080000	4.70190000	3.89330000

Table S3. DFT calculation result of **G@*P*-H6**.

Ag	-2.62290000	-1.34040000	1.69710000
Ag	0.61100000	-4.46190000	2.65950000
Ag	-0.76320000	1.65330000	-2.90550000
Ag	1.33180000	-0.96940000	-2.88260000
Ag	2.85470000	-4.18330000	0.30610000
Ag	-0.23990000	3.62270000	0.32270000
Ag	-1.71120000	-3.09390000	4.78530000
Ag	4.36080000	-2.21750000	-2.13830000
Ag	-3.10810000	1.42260000	-0.14200000
Ag	4.01250000	0.70070000	-3.93810000
S	-1.46340000	-3.44250000	1.51710000
S	-2.81980000	2.83400000	-2.16190000
S	1.87180000	-3.16560000	-1.91790000
S	-4.28960000	0.46130000	1.74960000
S	1.13680000	0.99670000	-4.29330000
O	6.01180000	-3.83650000	-1.72850000
O	2.80570000	-6.45410000	0.58280000
O	1.40480000	-6.59510000	2.38610000
O	5.30530000	-3.66730000	0.36190000
N	6.06220000	-4.23230000	-0.50960000
O	0.27610000	-3.91090000	4.92680000
O	6.84010000	-5.14870000	-0.18130000
O	3.78230000	-0.81350000	-5.65730000
O	-3.79810000	-2.31470000	4.75430000
C	3.16050000	-10.20320000	3.51970000
H	2.94380000	-10.65150000	4.49240000
C	4.09390000	-10.81640000	2.67100000
O	4.18170000	-2.67960000	-4.52810000
N	3.47280000	-2.03110000	-5.36190000
C	4.36120000	-10.24240000	1.41770000
H	5.08070000	-10.72740000	0.75070000
N	6.16400000	-11.65660000	3.85560000

O	-5.71650000	-2.39500000	3.67840000
N	0.37360000	-4.70610000	7.23240000
C	2.53060000	-9.01800000	3.13850000
H	1.81210000	-8.52140000	3.79680000
C	2.83860000	-8.41150000	1.91040000
C	3.74970000	-9.04250000	1.04820000
H	3.98910000	-8.55170000	0.10100000
C	-0.09700000	0.29920000	0.13970000
C	-4.38060000	2.82400000	-3.12840000
N	-4.68820000	-2.99570000	4.02130000
O	-4.42050000	-4.17320000	3.73220000
C	2.28480000	-7.03380000	1.58520000
C	4.84850000	-12.02130000	3.13140000
H	5.15190000	-12.67130000	2.29540000
H	4.27070000	-12.61420000	3.85690000
O	2.48140000	-2.55780000	-5.90800000
C	0.62480000	0.35360000	-5.94390000
C	-5.85660000	-0.24160000	1.07300000
C	7.10210000	-10.93550000	2.92940000
H	7.28580000	-11.56260000	2.04640000
H	8.04040000	-10.74270000	3.46540000
H	6.64500000	-9.98390000	2.63590000
C	-2.60400000	-4.75030000	0.89800000
C	0.62920000	-4.96140000	5.79980000
C	1.60380000	-4.48340000	-3.17080000
C	5.89000000	-10.79540000	5.05520000
H	5.47340000	-9.83740000	4.72370000
H	6.84020000	-10.61330000	5.57370000
H	5.17850000	-11.31510000	5.71120000
C	6.79500000	-12.93680000	4.30300000
H	6.10450000	-13.43890000	4.99640000
H	7.74280000	-12.69570000	4.80570000
H	6.97000000	-13.57200000	3.42430000
C	2.11110000	-5.27740000	5.57930000
H	2.49140000	-5.98640000	6.32720000
H	2.70530000	-4.35370000	5.64590000
H	2.24250000	-5.70180000	4.57160000
C	1.02050000	-3.50010000	7.73870000
H	2.10790000	-3.54800000	7.56840000
H	0.84810000	-3.43350000	8.82800000
H	0.62450000	-2.57670000	7.25980000

C	-1.04160000	-4.70000000	7.57840000
H	-1.57520000	-3.77230000	7.25780000
H	-1.14930000	-4.77630000	8.67640000
H	-1.54140000	-5.56570000	7.11250000
O	-0.66100000	1.28310000	-0.50590000
O	0.78010000	-0.44620000	-0.47200000
O	-0.42060000	0.06690000	1.36880000
Ag	2.49280000	2.01900000	-1.34080000
Ag	-1.08460000	5.02240000	-2.41670000
Ag	0.68210000	-1.21470000	3.22090000
Ag	-1.32490000	1.75290000	3.01960000
Ag	-3.15470000	4.55370000	-0.30540000
Ag	0.07490000	-2.89360000	-0.25160000
Ag	1.30440000	3.69090000	-4.48720000
Ag	-4.53770000	3.10210000	2.33520000
Ag	2.97620000	-0.89440000	0.53240000
Ag	-3.74000000	0.02200000	4.47510000
S	1.27580000	4.08270000	-1.51980000
S	2.55180000	-2.48660000	2.30930000
S	-1.97250000	3.86390000	2.02280000
S	4.16840000	0.23180000	-1.29940000
S	-1.07520000	-0.14800000	4.56130000
O	-5.99830000	4.85180000	1.78070000
O	-3.32120000	6.80220000	-0.22450000
O	-1.77270000	7.21080000	-1.85840000
O	-5.64760000	4.42140000	-0.36330000
N	-6.18030000	5.14930000	0.54630000
O	-0.30810000	5.22360000	-4.57520000
O	-6.87680000	6.14140000	0.24610000
O	-3.22840000	2.35270000	6.36020000
O	3.60610000	3.15000000	-4.70300000
C	-3.52050000	10.93670000	-2.60770000
H	-2.99660000	11.74590000	-3.12500000
C	-4.90890000	11.02470000	-2.41110000
O	-4.53900000	2.19320000	4.59220000
N	-3.83050000	2.93220000	5.43490000
C	-5.57290000	10.01370000	-1.70250000
H	-6.65150000	10.09420000	-1.53930000
N	-6.10300000	11.92670000	-4.44900000
O	5.20490000	2.84660000	-3.23590000
N	-1.38160000	4.46120000	-6.64890000

C	-2.82440000	9.81530000	-2.16170000
H	-1.75340000	9.70930000	-2.34740000
C	-3.49590000	8.78200000	-1.48920000
C	-4.87080000	8.90320000	-1.22780000
H	-5.37350000	8.10340000	-0.67450000
C	4.02510000	-2.57950000	3.40270000
N	4.41850000	3.65700000	-3.82350000
O	4.40480000	4.86920000	-3.56560000
C	-2.78520000	7.49170000	-1.14660000
C	-5.68320000	12.16530000	-2.98100000
H	-6.62270000	12.32960000	-2.43360000
H	-5.09970000	13.09960000	-2.99110000
O	-3.80190000	4.16090000	5.24520000
C	-0.40670000	0.44690000	6.16940000
C	5.74230000	0.94500000	-0.66020000
C	-6.90830000	10.66480000	-4.57960000
H	-7.78720000	10.72750000	-3.92310000
H	-7.21080000	10.55470000	-5.62980000
H	-6.28130000	9.81550000	-4.28500000
C	2.34920000	5.48920000	-1.00010000
C	-1.20230000	5.50870000	-5.60960000
C	-1.63660000	5.36220000	3.03650000
C	-4.90120000	11.84070000	-5.34500000
H	-4.30360000	10.96570000	-5.06380000
H	-5.24950000	11.71860000	-6.37930000
H	-4.30820000	12.75780000	-5.23570000
C	-6.94020000	13.09490000	-4.86380000
H	-6.33600000	14.00730000	-4.75760000
H	-7.23980000	12.94770000	-5.91220000
H	-7.82290000	13.13940000	-4.20840000
C	-2.56800000	5.89680000	-5.03210000
H	-3.30040000	6.10070000	-5.82990000
H	-2.95450000	5.07870000	-4.40130000
H	-2.46090000	6.77890000	-4.38150000
C	-1.89760000	3.20080000	-6.13220000
H	-2.88940000	3.34760000	-5.67660000
H	-2.00850000	2.48650000	-6.96770000
H	-1.23090000	2.73950000	-5.36670000
C	-0.16190000	4.23540000	-7.40810000
H	0.64200000	3.72140000	-6.81950000
H	-0.38220000	3.60360000	-8.28680000

H	0.24400000	5.20170000	-7.75330000
H	0.03800000	-5.88010000	5.57970000
H	-0.82060000	6.37820000	-6.19980000
H	-4.28730000	3.50270000	-3.98760000
H	-5.18910000	3.16520000	-2.46780000
H	-4.57730000	1.80260000	-3.48460000
H	0.37030000	1.21470000	-6.57810000
H	1.48310000	-0.20430000	-6.34510000
H	-0.24130000	-0.31110000	-5.83550000
H	-6.54260000	0.59440000	0.87370000
H	-6.27500000	-0.91670000	1.83160000
H	-5.65300000	-0.79100000	0.14550000
H	-2.01180000	-5.65600000	0.70830000
H	-3.35240000	-4.93410000	1.68210000
H	-3.09350000	-4.41730000	-0.02780000
H	2.26460000	-4.26160000	-4.02110000
H	1.85550000	-5.44940000	-2.71060000
H	0.55330000	-4.47340000	-3.49010000
H	3.79490000	-3.25430000	4.23810000
H	4.86320000	-2.97000000	2.80850000
H	4.25930000	-1.57670000	3.78520000
H	-0.22160000	-0.43170000	6.80460000
H	-1.15710000	1.10690000	6.62620000
H	0.53220000	0.99120000	6.00270000
H	6.48630000	0.13760000	-0.61790000
H	6.06240000	1.73830000	-1.34800000
H	5.57060000	1.35330000	0.34510000
H	1.72430000	6.39440000	-0.99890000
H	3.16230000	5.58200000	-1.73250000
H	2.75080000	5.30450000	0.00520000
H	-2.27720000	5.31110000	3.92700000
H	-1.89180000	6.23710000	2.42080000
H	-0.57620000	5.38340000	3.32140000
O	-8.72750000	15.38130000	-2.06600000
O	-7.48840000	6.61910000	-4.10070000
O	-9.62040000	9.91830000	-7.64550000
O	-0.71920000	10.47300000	-5.40930000
O	-3.07470000	14.76290000	-7.65450000
O	-5.43670000	12.48040000	0.88300000
C	-9.52980000	15.96550000	-3.09000000
H	-9.22950000	17.02240000	-3.13670000

H	-9.34700000	15.48490000	-4.07110000
H	-10.60630000	15.89520000	-2.84760000
C	-8.95380000	14.05380000	-1.76950000
C	-8.22830000	13.53070000	-0.67700000
C	-8.37820000	12.16920000	-0.36060000
H	-7.80360000	11.76540000	0.47820000
C	-9.24330000	11.35620000	-1.08620000
C	-10.00180000	11.90540000	-2.13340000
C	-9.85630000	13.24570000	-2.48960000
H	-10.46440000	13.65590000	-3.30030000
C	-9.53980000	9.87830000	-0.84010000
H	-8.93920000	9.44390000	-0.02630000
C	-11.04330000	9.81300000	-0.57180000
C	-11.66460000	9.28140000	0.55500000
H	-13.56060000	8.89840000	1.52700000
C	-13.06590000	9.31860000	0.64610000
H	-11.06530000	8.83500000	1.35560000
C	-13.82990000	9.87920000	-0.38280000
H	-13.79110000	10.85490000	-2.32990000
C	-13.20030000	10.41350000	-1.51930000
H	-14.92150000	9.90370000	-0.30420000
C	-11.81290000	10.37580000	-1.60710000
C	-10.96310000	10.90410000	-2.76140000
H	-11.56630000	11.33570000	-3.57620000
C	-10.09760000	9.73580000	-3.21480000
C	-10.02910000	9.20090000	-4.49620000
H	-10.64470000	9.60510000	-5.30670000
C	-9.15710000	8.14110000	-4.78280000
C	-8.34990000	7.61670000	-3.74370000
C	-8.45390000	8.11990000	-2.42840000
H	-7.87690000	7.67680000	-1.61230000
C	-9.31960000	9.18480000	-2.18360000
C	-6.66480000	6.05130000	-3.06050000
H	-6.01280000	6.81680000	-2.60800000
H	-7.26920000	5.58700000	-2.26700000
H	-6.04890000	5.29070000	-3.55890000
C	-9.07820000	7.58720000	-6.18880000
H	-10.06310000	7.70450000	-6.67070000
H	-8.83920000	6.51170000	-6.15240000
C	-9.96290000	11.06780000	-8.41300000
H	-9.37890000	11.95360000	-8.09290000

H	-9.79680000	10.89540000	-9.49220000
H	-11.03210000	11.24540000	-8.22740000
C	-8.31960000	9.46750000	-7.73430000
C	-8.02120000	8.29120000	-7.01140000
C	-6.70920000	7.79530000	-7.04730000
H	-6.47810000	6.89240000	-6.47480000
C	-5.72500000	8.43730000	-7.79200000
C	-6.03670000	9.59560000	-8.51880000
C	-7.32870000	10.12320000	-8.49120000
H	-7.56030000	11.01320000	-9.08150000
C	-4.84910000	10.13610000	-9.31190000
H	-5.08830000	11.04370000	-9.88850000
C	-4.37630000	8.98000000	-10.19390000
C	-4.21420000	8.99770000	-11.57620000
H	-3.58330000	7.85850000	-13.30910000
C	-3.72120000	7.85150000	-12.22310000
H	-4.46060000	9.89760000	-12.15030000
C	-3.39330000	6.70900000	-11.48680000
H	-3.29070000	5.80740000	-9.50220000
C	-3.56060000	6.69070000	-10.09180000
H	-2.99790000	5.82520000	-11.99690000
C	-4.05550000	7.82630000	-9.45650000
C	-4.27770000	7.99370000	-7.95710000
H	-4.03600000	7.08710000	-7.38990000
C	-3.43200000	9.19420000	-7.55590000
C	-3.73370000	10.35000000	-8.29190000
C	-3.00740000	11.51510000	-8.06710000
H	-3.20780000	12.42110000	-8.64760000
C	-1.98500000	11.55130000	-7.10390000
C	-1.71160000	10.38460000	-6.35210000
C	-2.43450000	9.19900000	-6.58660000
H	-2.21590000	8.28660000	-6.03420000
C	-0.27690000	9.23690000	-4.81280000
H	0.57640000	9.50820000	-4.17550000
H	-1.06350000	8.77120000	-4.19660000
H	0.05090000	8.52130000	-5.58590000
C	-1.19690000	12.82570000	-6.88930000
H	-0.18940000	12.57790000	-6.51890000
H	-1.08570000	13.34350000	-7.85620000
C	-3.98500000	15.76290000	-8.10520000
H	-3.62090000	16.77830000	-7.86360000

H	-4.04480000	15.64240000	-9.19660000
H	-4.98960000	15.61970000	-7.66100000
C	-2.80810000	14.71570000	-6.30460000
C	-1.86570000	13.74720000	-5.89380000
C	-1.57420000	13.63430000	-4.52520000
H	-0.84850000	12.87780000	-4.20990000
C	-2.18300000	14.47070000	-3.59330000
C	-3.09750000	15.44610000	-4.01880000
C	-3.42870000	15.56630000	-5.36780000
H	-4.12960000	16.34200000	-5.68550000
C	-3.60760000	16.32520000	-2.87920000
H	-4.32230000	17.09380000	-3.21300000
C	-2.35600000	16.91770000	-2.23160000
C	-2.07680000	18.26900000	-2.04800000
H	-0.64080000	19.70020000	-1.27950000
C	-0.87090000	18.64040000	-1.42920000
H	-2.78970000	19.03040000	-2.38200000
C	0.03430000	17.66380000	-1.00440000
H	0.45750000	15.53320000	-0.85750000
C	-0.24980000	16.30040000	-1.18910000
H	0.97150000	17.96260000	-0.52290000
C	-1.44530000	15.93470000	-1.80240000
C	-1.91910000	14.51210000	-2.09160000
H	-1.20420000	13.74440000	-1.75890000
C	-3.28510000	14.38640000	-1.42760000
C	-3.65540000	13.41650000	-0.50210000
H	-2.93740000	12.65380000	-0.19860000
C	-4.97020000	13.41650000	0.00080000
C	-5.89220000	14.41010000	-0.40090000
C	-5.48890000	15.37780000	-1.33230000
H	-6.21270000	16.13640000	-1.64680000
C	-4.19620000	15.36620000	-1.85170000
C	-4.50960000	11.47870000	1.33000000
H	-3.66620000	11.94430000	1.87050000
H	-5.07960000	10.83450000	2.01300000
H	-4.13760000	10.87770000	0.48330000
C	-7.30580000	14.41010000	0.13810000
H	-7.30470000	14.05020000	1.17970000
H	-7.68530000	15.44430000	0.13600000
O	0.73020000	-12.03940000	4.69760000
O	8.60950000	-15.48470000	1.40300000

O	5.85640000	-11.94980000	-1.47530000
O	10.18740000	-9.24350000	5.92620000
O	6.46800000	-6.31330000	3.67480000
O	4.34130000	-13.89340000	8.23460000
C	-0.04480000	-11.46670000	3.63480000
H	-0.67000000	-10.69660000	4.10940000
H	0.60730000	-10.99570000	2.87860000
H	-0.68560000	-12.23320000	3.16150000
C	1.55260000	-13.08950000	4.38040000
C	2.19740000	-13.72560000	5.46560000
C	3.05340000	-14.80900000	5.20660000
H	3.53900000	-15.30200000	6.05540000
C	3.25230000	-15.26220000	3.90510000
C	2.60620000	-14.61900000	2.83830000
C	1.76020000	-13.53610000	3.06030000
H	1.27300000	-13.04620000	2.21560000
C	4.10300000	-16.44870000	3.46300000
H	4.59920000	-16.96000000	4.30250000
C	3.16140000	-17.36460000	2.68300000
C	2.89990000	-18.70470000	2.94960000
H	1.76840000	-20.45180000	2.34490000
C	1.98350000	-19.39830000	2.14140000
H	3.39760000	-19.20820000	3.78490000
C	1.34450000	-18.74990000	1.08100000
H	1.11300000	-16.88540000	-0.01740000
C	1.61170000	-17.39750000	0.81170000
H	0.62990000	-19.29660000	0.45710000
C	2.52010000	-16.71210000	1.61380000
C	2.92380000	-15.24420000	1.48410000
H	2.42830000	-14.73330000	0.64470000
C	4.44420000	-15.23730000	1.37190000
C	5.20690000	-14.68140000	0.34770000
H	4.72710000	-14.16820000	-0.49150000
C	6.60750000	-14.76910000	0.36310000
C	7.23480000	-15.43110000	1.44220000
C	6.46940000	-15.99670000	2.48100000
H	6.94420000	-16.53020000	3.30780000
C	5.08130000	-15.89200000	2.43600000
C	9.26150000	-16.23940000	2.42270000
H	9.08640000	-15.79870000	3.42360000
H	8.92230000	-17.29160000	2.42250000

H	10.33470000	-16.19850000	2.18580000
C	7.42010000	-14.16540000	-0.76060000
H	6.81430000	-14.18490000	-1.68120000
H	8.32130000	-14.77660000	-0.93310000
C	5.17170000	-10.88030000	-2.14120000
H	4.81630000	-10.11940000	-1.42620000
H	5.82480000	-10.40210000	-2.89330000
H	4.30730000	-11.34580000	-2.63460000
C	7.05520000	-11.64530000	-0.87140000
C	7.84820000	-12.74360000	-0.47040000
C	9.06540000	-12.49360000	0.18520000
H	9.67570000	-13.34880000	0.49480000
C	9.49110000	-11.19220000	0.42560000
C	8.70730000	-10.11050000	-0.00710000
C	7.49320000	-10.32300000	-0.65360000
H	6.90060000	-9.46530000	-0.98250000
C	9.32430000	-8.75480000	0.32340000
H	8.71440000	-7.90250000	-0.01450000
C	10.72140000	-8.77840000	-0.29270000
C	11.23310000	-7.86080000	-1.20420000
H	12.96260000	-7.30690000	-2.38480000
C	12.54910000	-8.02260000	-1.66830000
H	10.61480000	-7.02420000	-1.54840000
C	13.33300000	-9.08960000	-1.21860000
H	13.42240000	-10.85710000	0.05110000
C	12.81260000	-10.01620000	-0.30040000
H	14.35790000	-9.20570000	-1.58600000
C	11.50780000	-9.85470000	0.15770000
C	10.76850000	-10.76250000	1.13870000
H	11.37840000	-11.61480000	1.48010000
C	10.30150000	-9.86120000	2.27790000
C	9.53360000	-8.77540000	1.83460000
C	9.04470000	-7.84820000	2.74820000
H	8.45970000	-6.98840000	2.40760000
C	9.28720000	-8.00040000	4.12260000
C	10.02030000	-9.12500000	4.56170000
C	10.54570000	-10.05110000	3.63760000
H	11.14900000	-10.90240000	3.96510000
C	10.96700000	-10.34190000	6.38890000
H	11.00800000	-10.24370000	7.48350000
H	10.49730000	-11.30870000	6.11790000

H	11.99230000	-10.31250000	5.97640000
C	8.75640000	-6.98340000	5.10860000
H	9.43420000	-6.91790000	5.97570000
H	8.73440000	-5.99760000	4.61440000
C	5.34060000	-6.01140000	2.83030000
H	4.60230000	-5.38830000	3.36340000
H	5.74220000	-5.46320000	1.96930000
H	4.86130000	-6.94370000	2.49260000
C	6.22810000	-6.99100000	4.83270000
C	7.36900000	-7.33420000	5.59900000
C	7.19060000	-8.00180000	6.81740000
H	8.07670000	-8.25910000	7.40790000
C	5.91300000	-8.31730000	7.27620000
C	4.79020000	-7.97620000	6.50720000
C	4.93540000	-7.32510000	5.28480000
H	4.05420000	-7.06180000	4.70090000
C	3.47000000	-8.37110000	7.17050000
H	2.58760000	-8.11600000	6.56700000
C	3.48660000	-7.68240000	8.53570000
C	2.54770000	-6.77540000	9.01830000
H	1.99970000	-5.51450000	10.68510000
C	2.72820000	-6.23550000	10.30250000
H	1.69600000	-6.45130000	8.40480000
C	3.83100000	-6.59780000	11.08110000
H	5.65630000	-7.78120000	11.18180000
C	4.78100000	-7.50480000	10.58280000
H	3.96370000	-6.16600000	12.07880000
C	4.60750000	-8.03800000	9.31030000
C	5.55430000	-9.00060000	8.59310000
H	6.43740000	-9.26370000	9.19690000
C	4.71640000	-10.20910000	8.19980000
C	4.99920000	-11.54280000	8.48550000
H	5.89120000	-11.79240000	9.06530000
C	4.12150000	-12.54650000	8.02910000
C	2.94220000	-12.20910000	7.33150000
C	2.69220000	-10.85610000	7.04900000
H	1.77840000	-10.59750000	6.50550000
C	3.57940000	-9.86370000	7.45250000
C	5.43410000	-14.25020000	9.07950000
H	5.33000000	-13.79330000	10.08110000
H	5.39760000	-15.34580000	9.16730000

H	6.40200000	-13.94380000	8.63760000
C	1.95800000	-13.26940000	6.88810000
H	2.02790000	-14.14340000	7.55630000
H	0.93880000	-12.85850000	6.97320000

8. References

1. B. Delley, *J. Chem. Phys.*, 1990, **92**, 508−517.

2. B. Delley, *J. Chem. Phys.*, 2000, **113**, 7756−7764.

3. J. P. Perdew, K. Burke and M. Ernzerhof, *Phys. Rev. Lett.*, 1996, **77**, 3865−3868.

4. S. Yang, S. Chen, L. Xiong, C. Liu, H. Yu, S. Wang, N. L. Rosi, Y. Pei and M. Zhu, *J. Am. Chem. Soc.*, 2018, **140**, 10988−10994.
